# Comparative Analysis of Salivary Gland Transcriptomes of *Phlebotomus orientalis* Sand Flies from Endemic and Non-endemic Foci of Visceral Leishmaniasis

**DOI:** 10.1371/journal.pntd.0002709

**Published:** 2014-02-27

**Authors:** Michaela Vlkova, Michal Sima, Iva Rohousova, Tatiana Kostalova, Petra Sumova, Vera Volfova, Erin L. Jaske, Kent D. Barbian, Teshome Gebre-Michael, Asrat Hailu, Alon Warburg, Jose M. C. Ribeiro, Jesus G. Valenzuela, Ryan C. Jochim, Petr Volf

**Affiliations:** 1 Department of Parasitology, Faculty of Science, Charles University, Prague, Czech Republic; 2 Genomics Unit, Research Technologies Section, Rocky Mountain Laboratories, Hamilton, Montana, United States of America; 3 Aklilu Lemma Institute of Pathobiology, Addis Ababa University, Addis Ababa, Ethiopia; 4 Department of Microbiology, Immunology & Parasitology, Faculty of Medicine, Addis Ababa University, Addis Ababa, Ethiopia; 5 Department of Parasitology, The Kuvin Centre for the Study of Infectious and Tropical Diseases, Hadassah Medical School, The Hebrew University of Jerusalem, Jerusalem, Israel; 6 Vector Biology Section, Laboratory of Malaria and Vector Research, National Institute of Allergy and Infectious Diseases, National Institutes of Health, Rockville, Maryland, United States of America; 7 Vector Molecular Biology Section, Laboratory of Malaria and Vector Research, National Institute of Allergy and Infectious Diseases, National Institutes of Health, Rockville, Maryland, United States of America; University of Notre Dame, United States of America

## Abstract

**Background:**

In East Africa, *Phlebotomus orientalis* serves as the main vector of *Leishmania donovani*, the causative agent of visceral leishmaniasis (VL). *Phlebotomus orientalis* is present at two distant localities in Ethiopia; Addis Zemen where VL is endemic and Melka Werer where transmission of VL does not occur. To find out whether the difference in epidemiology of VL is due to distant compositions of *P. orientalis* saliva we established colonies from Addis Zemen and Melka Werer, analyzed and compared the transcriptomes, proteomes and enzymatic activity of the salivary glands.

**Methodology/Principal Findings:**

Two cDNA libraries were constructed from the female salivary glands of *P. orientalis* from Addis Zemen and Melka Werer. Clones of each *P. orientalis* library were randomly selected, sequenced and analyzed. In *P. orientalis* transcriptomes, we identified members of 13 main protein families. Phylogenetic analysis and multiple sequence alignments were performed to evaluate differences between the *P. orientalis* colonies and to show the relationship with other sand fly species from the subgenus *Larroussius*. To further compare both colonies, we investigated the humoral antigenicity and cross-reactivity of the salivary proteins and the activity of salivary apyrase and hyaluronidase.

**Conclusions:**

This is the first report of the salivary components of *P. orientalis*, an important vector sand fly. Our study expanded the knowledge of salivary gland compounds of sand fly species in the subgenus *Larroussius*. Based on the phylogenetic analysis, we showed that *P. orientalis* is closely related to *Phlebotomus tobbi* and *Phlebotomus perniciosus*, whereas *Phlebotomus ariasi* is evolutionarily more distinct species. We also demonstrated that there is no significant difference between the transcriptomes, proteomes or enzymatic properties of the salivary components of Addis Zemen (endemic area) and Melka Werer (non-endemic area) *P. orientalis* colonies. Thus, the different epidemiology of VL in these Ethiopian foci cannot be attributed to the salivary gland composition.

## Author Summary

*Phlebotomus orientalis* is the vector of visceral leishmaniasis (VL) caused by *Leishmania donovani* in Northeast Africa. Immunization with sand fly saliva or with individual salivary proteins has been shown to protect against leishmaniasis in different hosts, warranting the intensive study of salivary proteins of sand fly vectors. In our study, we characterize the salivary compounds of *P. orientalis*, thereby broadening the repertoire of salivary proteins of sand fly species belonging to the subgenus *Larroussius*. In order to find out whether there is any connection between the composition of *P. orientalis* saliva and the epidemiology of VL in two distinct Ethiopian foci, Addis Zemen and Melka Werer, we studied the transcriptomes, proteomes, enzymatic activities, and the main salivary antigens in two *P. orientalis* colonies originating from these areas. We did not detect any significant difference between the saliva of female sand flies originating in Addis Zemen (endemic area) and Melka Werer (non-endemic area). Therefore, the different epidemiology of VL in these Ethiopian foci cannot be related to the distant salivary gland protein composition. Identifying the sand fly salivary gland compounds will be useful for future research focused on characterizing suitable salivary proteins as potential anti-*Leishmania* vaccine candidates.

## Introduction

Protozoan parasites belonging to the genus *Leishmania* are the pathogenic agents causing a broad range of diseases commonly known as leishmaniasis. Sand fly vectors (Diptera: Phlebotominae) spread leishmaniasis among the vertebrate hosts during the bloodfeeding when infected sand fly females eject parasites into the wound along with their saliva. Salivary compounds possess powerful anti-hemostatic and immunomodulatory properties (reviewed in [1]); nonetheless, the salivary proteins are highly antigenic. As the repeated exposure to sand fly bites was shown to be protective against leishmaniasis (e.g. [2]), the immune profiles elicited by single salivary proteins are of major scientific interest.

To date, the intensive investigation of salivary proteins in certain sand fly species has allowed the generation of individual recombinant salivary proteins that have been employed as reliable markers of exposure to sand fly bites [3]–[5] or as the protective agent against cutaneous and visceral leishmaniases (CL and VL, respectively) under laboratory conditions [6]–[13]. However, most of the experiments were performed using New World VL vector *Lutzomyia longipalpis*. As the composition of salivary glands and the protective effect conferred by sand fly saliva is species-specific [14]–[19], it is vital to continue with detailed characterization of the salivary proteins with a special focus on sand fly species causing lethal VL.

*Phlebotomus orientalis* is a member of the subgenus *Larroussius* and represents the main sand fly species transmitting *Leishmania donovani* within the countries of East Africa (reviewed in [20]) as well as in Saudi Arabia [21] and Yemen [22]. At two distinct localities in Ethiopia, Addis Zemen and Melka Werer, we observed different epidemiology of VL, although *P. orientalis* was present in both places. While in Addis Zemen, human VL caused by *Le. donovani* with high mortality rate was reported [23], Melka Werer is considered to be a non-endemic area with no human cases. A recently published study compared various molecular aspects of colonies from both foci and showed that the susceptibility of Addis Zemen and Melka Werer colonies to *Le. donovani* infection was identical [24]. As Warburg et al. described the possible connection of the salivary gland composition with varying pathologies of CL [25] and sand fly saliva is known to play a crucial role in transmission of *Leishmania* spp. (e.g. [2]), we hypothesized that the composition of salivary glands may explain the different epidemiology in these Ethiopian foci. Therefore, we studied the transcriptomes, proteomes and the enzymatic activities (apyrase and hyaluronidase) in the saliva of female sand flies from Addis Zemen (VL endemic) and from Melka Werer (non-endemic). Furthermore, we characterized the main salivary antigens in both colonies and determined the level of glycosylation of *P. orientalis* salivary proteins. Importantly, we compared our data with other sand fly species from the subgenus *Larroussius*, whose cDNA libraries have already been constructed [26]–[28], and used sequences of the New World sand fly species *L. longipalpis* as an outgroup.

## Methods

### Ethics statement

BALB/c mice were maintained and handled in the animal facility of Charles University in Prague in accordance with institutional guidelines and Czech legislation (Act No. 246/1992 coll. on Protection of Animals against Cruelty in present statutes at large). The experiments were approved by the Committee on the Ethics of Animal Experiments of the Charles University in Prague (Permit Number: 24773/2008-10001) and were performed under the Certificate of Competency (Registration Numbers: CZU 934/05, CZU 307/09) in accordance with the Examination Order approved by Central Commission for Animal Welfare of the Czech Republic.

### Sand flies and salivary gland dissections

Two colonies of *P. orientalis* were established; one from a non-endemic lowland area in central Ethiopia, Melka Werer (MW) (altitude of 800 m), the later one from an endemic focus of VL in the highlands of Northwest Ethiopia, Addis Zemen (AZ) (altitude of 1800–2000 m), and then transferred to Czech Republic. Both sand fly colonies were kept in the insectary of Charles University in Prague and were reared under standard conditions as described in [29]. For the experiments, the sand flies from F5–F6 generation were used. Salivary glands of 1-day old adult females were dissected; mRNA was extracted and stored in RNA later (Ambion). For proteome analysis, western blot, affinity blot, and hyaluronidase assay, salivary glands from 5- to 8-day old *P. orientalis* adult females were dissected and stored in Tris buffer (20 mM Tris, 150 mM NaCl, pH 7.7). For the apyrase assay, 8-day old adult female salivary glands were dissected into Tris buffer containing 0.005% Triton X-100 and stored at −80°C.

### Construction of salivary gland cDNA libraries

Salivary gland mRNA was isolated separately from 45 pairs each of MW and AZ glands using Micro-FastTrack mRNA isolation kit (Invitrogen). Both cDNA libraries were constructed following the manufacturer's instructions for SMART cDNA Library Construction Kit (BD Clontech) with some modifications as described in [30]. Each library was fractionated into large, medium, and small cDNA fragments. Gigapack III Gold Packaging Extract (Stratagene) was used for packaging the phage. Both libraries were then plated by infecting log-phase XL-1 blue *Escherichia coli* (Clontech). Transfected plaques were randomly selected and a PCR reaction with vector primers flanking the inserted cDNA was made. The presence of recombinants was checked by visualization the PCR products on 1.1% agarose gel with SYBR Safe (Invitrogen). Inserts were sequenced as previously described [31] using a ABI 3730XL DNA Sequencer (Applied Biosystems).

### Bioinformatics

Detailed description of the bioinformatics analysis can be found elsewhere [28]. Briefly, expression sequence tags (ESTs) were analyzed using a customized program based on the Phred algorithm [32], [33]. Sequences with Phred quality scores lower than 25 were removed, as well as vector sequences and primers. Resulting sequences were grouped based on nucleotide homology of 90% identity over 100 residues and aligned into consensus transcript sequences (contigs) using the CAP3 sequence assembly program. BLAST programs were used to compare contigs and singletons (contigs with a single sequence) to the non-redundant protein database of the NCBI, the Gene Ontology database (GO) [34], to COG conserved domains database [35], Protein Family database (Pfam) [36], SimpleModular Architecture Tool database (SMART) [37], and to rRNA Nucleotide Sequences, and Mitochondrial and Plastid Sequence (MITPLA) databases available from NCBI. The three frame translations of each dataset were submitted to the SignalP server [38] to find signal sequences. The grouped and assembled sequences, BLAST results, and SignalP results, combined by dCAS software [39] in an Excel spreadsheet, were manually verified and annotated. N- and O-Glycosylation sites on the proteins were predicted using NetNGlyc 1.0 and NetOGlyc 3.1 software (www.cbs.dtu.dk/services/NetNGlyc, www.cbs.dtu.dk/services/NetOGlyc) [40].

### Phylogenetic analysis

Protein sequences were aligned using ClustalX (version 2.0) [41] and manually refined in BioEdit 7.1.3.0 editing software. For each alignment, best substitution matrix was determined by ProtTest software 2.0 [42]. This matrix was subsequently used by TREE-PUZZLE 5.2 [43] to reconstruct maximum likelihood phylogenetic trees from the protein alignments using quartet puzzling with 1000 puzzling steps in each phylogenetic analysis. Resulting trees were visualized in MEGA 4 [44].

### Proteome analysis

For mass spectrometry analysis, salivary glands of both AZ and MW *P. orientalis* colonies were dissolved in non-reducing sample buffer and electrophoretically separated in 12.5% SDS gel. Proteins within the gel were visualized by staining with Coomassie Brilliant Blue R-250 (Serva). The individual bands were cut and incubated with 10 mM dithiothreitol (Sigma) and then treated with 55 mM iodoacetamide (Sigma). Washed and dried bands were digested with trypsin (Promega). The tryptic peptides were separated by liquid chromatography using an Ultimate 3000 HPLC system (Dionex). The peptide samples diluted in 0.3% trichloroacetic acid (TCA) with 10% acetonitrile (ACN) were loaded onto a PepMap 100 C18 RP column (Dionex) at a flow rate of 300 nl per minute. The peptides were eluted by a 45-min linear gradient of 5–80% (v/v) ACN in 0.1% (v/v) TCA over a period of 20 min. The eluent was mixed 1∶3 with matrix solution (20 mg/ml a-cyano-4-hydroxycinnamic acid in 80% ACN) and subsequently spotted onto MALDI target plates using a Probot microfraction collector (Dionex). Spectra were acquired on 4800 Plus MALDI TOF/TOF analyzer (Applied Biosystems/MDS Sciex) equipped with a Nd: YAG laser (355 nm, firing rate 200 Hz) as described in detail in [28].

### Hyaluronidase activity analysis

Hyaluronidase activity in salivary glands of both *P. orientalis* colonies was quantified using a sensitive assay in microtitration plates coupled with biotinylated hyaluronic acid (bHA). Salivary glands were homogenized by three freeze-thaw cycles and salivary gland extract (SGE) was obtained by centrifugation at 17000 g (5 min, 2°C). Biotinylated HA, prepared as described in [45], was immobilized onto Covalink NH microtiter plates (NUNC) using the method by Frost and Stern [46] modified by [28] at a final concentration of 1 µg/well bHA. The plates were incubated overnight at 4°C and washed three times in PBS, pH 7.2 containing 2 M NaCl and 50 mM MgSO_4_. The plates with immobilized bHA were coated for 45 min with 1% BSA in PBS, then washed and equilibrated with assay buffer (0.1 M acetate buffer, pH 5.0, 0.1 M NaCl, 0.1% Triton X-100) to adjust the pH for optimum sand fly salivary hyaluronidase activity. Four SGE samples for each colony were pipetted into the plates in triplicate at a final concentration of 0.5 salivary gland per well and incubated for 45 min at 37°C. To obtain a standard curve ranging from 0.5 to 7.8×10^3^ rTRU, hyaluronidase from bovine testes (Sigma), at a concentration of 0.01 TRU/µl, was diluted by two-fold serial dilution in 0.1 M acetate buffer, pH 4.5, 0.1 M NaCl, 0.1% Triton X-100. Wells without bHA or enzyme were used as controls. The reaction was terminated by the addition of 200 µl/well of 6 M guanidine. After washing, avidin-peroxidase (Sigma, 2 µg/ml) was added at a final concentration of 0.2 µg/well and incubated for 30 min at room temperature. Color reaction was developed with o-phenylenediamine substrate in 0.1 M citrate-phosphate buffer, pH 5.5. Absorbance was measured at 492 nm using Infinite M 200 fluorometer (Schoeller Instruments). Raw data were evaluated by Measurement Parameters Editor Magellan 6 (Tecan) and the standard curve created using a 4-parameter logistic fit.

### Apyrase activity analysis

Apyrase activity was determined using the Fiske and Subbarow method for measuring inorganic phosphate (Pi) released from ADP or ATP [47], with some modifications. Salivary glands were homogenized by one freeze-thaw cycle combined with a mechanical homogenization. Two µl of salivary gland homogenate (SGH) diluted 1∶25 in assay buffer (50 mM TRIS 150 mM NaCl, pH 8.5 with 5 mM CaCl_2_ or 5 mM MgCl_2_) were mixed in wells with 78 µl of assay buffer and 20 µl of substrate to obtain a final concentration of 2 mM ATP or ADP and 1/25 of gland pair per well. SGH samples were pipetted into the microtiter plate in series of six. Wells containing only assay buffer were used as negative controls. Plates were incubated for 15 min at 37°C. Then the enzymatic reaction was stopped by addition of 25 µl of 1.25% ammonium molybdate in 1.25 M sulfuric acid and 5 µl of Fiske-Subbarow reducer (25 mg/ml, F5428 Sigma) per well. The colorimetric reaction was read after 15 min by Tecan Infinite M 200 fluorometer (Schoeller Instruments) at 665 nm. The amount of Pi released from substrate was determined using potassium dihydrogen phosphate as a standard. The study of pH optimum was carried out within a range of pH 6.0–9.5. Salivary glands of *P. papatasi*, the species with previously described apyrase activity [48], were used as a positive control. Amount of proteins within SGHs was determined using Bio-Rad *DC* Protein Assay with BSA as a standard according to the manufacturer's instructions.

### Western blotting

Salivary glands of both *P. orientalis* colonies were separated by SDS-PAGE on 10% gel under non-reducing conditions using Mini-Protean III apparatus (Biorad). Salivary proteins were transferred from gel to nitrocellulose membrane (NC) by Semi-Phor equipment (Hoefer Scientific Instruments) and cut into strips. The strips were then blocked with 5% low fat dry milk in Tris-buffered saline with 0.05% Tween 20 (TBS-Tw) and subsequently incubated with BALB/c mice sera (AZ – mice bitten 18 times in a week interval; MW – mice bitten 17 times in a week interval), diluted 1∶100 in TBS-Tw, for 1 hour. After the washing with TBS-Tw, the strips were incubated for 1 hour with peroxidase-conjugated goat anti-mouse IgG (Serotec) diluted 1∶1000 in TBS-Tw. The chromogenic reaction was developed using a substrate solution containing diaminobenzidine and H_2_O_2_.

### Affinity blotting

Affinity blotting was performed using salivary glands from MW *P. orientalis* colony separated by SDS-PAGE as described above. After transfer, free binding sites on NC membrane were blocked with 5% bovine serum albumin in 20 mM TBS-Tw overnight at 4°C. The strips were then incubated for 1.5 hour on the shaker at room temperature with biotinylated lectins from *Dolichos biflorus* (DBA, Vector), *Glycine max* (SBA, Vector), *Ulex europaeus* (UEA-I, Vector), *Tetragonolobus purpureus* (LTA, Sigma), *Canavalia ensiformis* (ConA, Sigma), and *Pisum sativum* (PSA, Vector). Based on the preliminary experiments with different lectin concentrations, the lectins were diluted: 5 µg/ml, 10 µg/ml, 10 µg/ml, 0.2 µg/ml, 0.1 µg/ml and 10 µg/ml in TBS-Tw, respectively. To control the reaction specificity the aforementioned lectins were pre-incubated for 30 min with the appropriate saccharide inhibitors (Sigma) as follows: 0.25 M N-acetyl-D-galactosamine for DBA and SBA, 0.5 M L-fucose for UEA-I and LTA, 0.5 M methyl-α-D-mannopyranoside for ConA and PSA, and subsequently applied on the strips. After the washing with TBS-Tw, streptavidin-peroxidase (Sigma) was added to strips at a final concentration of 1 µg/ml and incubated for 1 h on the shaker at room temperature. The chromogenic reaction was developed as mentioned above.

## Results and Discussion

### Sequencing of *P. orientalis* salivary gland cDNA libraries

Two cDNA libraries were constructed from salivary glands of *P. orientalis* colonies originating in Addis Zemen and Melka Werer, Ethiopia. For each cDNA library, 940 clones were randomly selected and sequenced, which resulted in 835 and 749 high quality sequences from AZ and MW, respectively. Based on nucleotide homology, sequences were clustered into contigs, analyzed using the dCAS cDNA annotation software [39] and subsequently verified by manual annotation. From the AZ cDNA library, sequences were assembled into 263 contigs, where 185 of them were singletons (one sequence per contig). From the MW cDNA library, we obtained 242 contigs, including 171 singletons. In accordance with previously published cDNA libraries from sand fly salivary glands, the most abundant transcripts were those coding for putative salivary proteins (607 out of 835 in AZ; 567 out of 749 in MW). Of the nucleotide sequences encoding putative salivary proteins, 574 (AZ) and 506 (MW) salivary transcripts encoded a predicted signal peptide sequence. Those that did not possess sequences encoding a signal peptide were truncated at the 5′ end. Most of the contigs coding for putative salivary proteins were comprised of more than one sequence (averaging 7.14 sequences per contig in AZ and 6.23 in MW), whereas housekeeping proteins or proteins with unknown function were mostly represented by singletons. All obtained ESTs were deposited in the NCBI dbEST database under accession numbers JZ479238–JZ480094 for AZ colony and JZ480095–JZ480885 for MW colony.

Members of 13 main protein families were found among the putative salivary proteins of the two *P. orientalis* colonies: apyrase, yellow-related protein, antigen 5-related protein, odorant-binding proteins (D7-related and PpSP15-like proteins), hyaluronidase, endonuclease, phospholipase, pyrophosphatase, amylase, PpSP32-like protein, ParSP25-like protein, SP16-like protein, and Lufaxin (SP34-like protein). Detailed descriptions of each protein family are listed in the following paragraphs. Interestingly, we did not detect any sequences coding for adenosin deaminase in either *P. orientalis* cDNA library. Thus, we expect that *P. orientalis* saliva contains adenosin and ADP/AMP; leaving only *P. duboscqi*, *L. longipalpis*, and *L. intermedia* [49]–[52] as the sand fly species identified to produce adenosine deaminase, to date.

BLAST comparison of translated nucleotide sequences with the non-redundant (NR) protein database showed high similarity with salivary proteins of *P. perniciosus* and *P. tobbi* (both subgenus *Larroussius*). Sporadically, the best match was found with salivary proteins of *P. arabicus* (subgenus *Adlerius*) or *P. argentipes* (subgenus *Euphlebotomus*). Representative sequences of putative salivary proteins from both *P. orientalis* colonies that were deposited into NCBI GenBank database are listed in Table 1 and Table 2. Both tables include GenBank accession numbers, the predicted molecular weight, isoelectric point, best match to the NR database, the sand fly species with the highest homology, and presence in the proteome.

**Table 1:** Salivary gland transcripts of *Phlebotomus orientalis* – Addis Zemen colony.

Cluster	Sequence name	Accession number	Proteome	MW	pI	Best match to NR protein database		
						Accession number	Species	E-value
PorASP2	42 kDa yellow-related salivary protein	KC170933	Y	41.54	6.09	ABA43049	*Phlebotomus perniciosus*	0.0
PorASP4	42.6 kDa yellow-related salivary protein	KC170934	Y	42.31	8.07	ADJ54080	*Phlebotomus tobbi*	0.0
PorASP11	35.5 kDa salivary apyrase	KC170935	Y	35.53	9.95	ABB00906	*Phlebotomus perniciosus*	0.0
PorASP14	35.2 kDa salivary apyrase	KC170936	Y	35.08	8.99	ADJ54077	*Phlebotomus tobbi*	0.0
PorASP15	35.2 kDa salivary apyrase	KC170937	Y	35.33	9.16	ADJ54077	*Phlebotomus tobbi*	0.0
PorASP28	14.6 kDa PpSP15-like salivary protein	KC170938	Y	14.53	8.88	ADJ54089	*Phlebotomus tobbi*	2e-75
PorASP31	14.4 kDa PpSP15-like salivary protein	KC170939		14.32	8.73	ADJ54088	*Phlebotomus tobbi*	6e-77
PorASP37	14.9 kDa PpSP15-like salivary protein	KC170940	Y	14.91	8.77	ADJ54084	*Phlebotomus tobbi*	3e-73
PorASP40	3.7 kDa-like salivary protein	KC170941		3.93	9.16	ADJ54106	*Phlebotomus tobbi*	2e-07
PorASP46	27 kDa D7-related salivary protein	KC170942		26.68	6.36	ABA43052	*Phlebotomus perniciosus*	4e-151
PorASP48	27.1 kDa D7-related salivary protein	KC170943	Y	26.93	8.26	ADJ54095	*Phlebotomus tobbi*	9e-162
PorASP61	13.8 kDa PpSP15-like salivary protein	KC170944	Y	13.88	9.07	ADJ54086	*Phlebotomus tobbi*	1e-68
PorASP64	14.7 kDa PpSP15-like salivary protein	KC170945		14.70	7.99	ADJ54085	*Phlebotomus tobbi*	8e-62
PorASP68	5.0 kDa-like salivary protein	KC170946		4.89	9.84	ADJ54105	*Phlebotomus tobbi*	5e-15
PorASP74	28.8 kDa antigen 5-related salivary protein	KC170947	Y	28.78	8.94	ADJ54083	*Phlebotomus tobbi*	3e-151
PorASP76	30 kDa antigen 5-related salivary protein	KC170948	Y	28.78	8.94	ABA43055	*Phlebotomus perniciosus*	1e-179
PorASP80	30 kDa salivary phospholipase A2	KC170949		29.66	8.44	ABA43062	*Phlebotomus perniciosus*	0.0
PorASP86	24.53 kDa PpSP32-like salivary protein	KC170950		24.97	10.14	ADJ54102	*Phlebotomus tobbi*	2e-125
PorASP98	4.5 kDa-like salivary protein	KC170952		5.63	10.51	ADJ54097	*Phlebotomus tobbi*	3e-18
PorASP106	38.8 kDa ParSP25-like salivary protein	KC170953		27.61	4.72	ADJ54098	*Phlebotomus tobbi*	1e-140
PorASP112	salivary hyaluronidase	KC170958		37.22	6.50	ACS93505	*Phlebotomus arabicus*	1e-178
PorASP122	27 kDa D7-related salivary protein SP10	KC170954	Y	26.76	9.20	ABA43058	*Phlebotomus perniciosus*	6e-155
PorASP139	41 kDa salivary endonuclease	KC170955		41.66	9.27	ABA43064	*Phlebotomus perniciosus*	0.0
PorASP150	16 kDa salivary protein A	KC170956		16.04	5.04	ACS93506	*Phlebotomus arabicus*	1e-42
PorASP262	47 kDa pyrophosphatase-like salivary protein SP132	KC170959		32.88	7.18	ABA12155	*Phlebotomus argentipes*	8e-163

Putatively secreted salivary proteins from AZ *Phlebotomus orientalis* colony with the number of cluster, GenBank accession number, presence in proteome, putative mature protein features (MW- molecular weight, pI- isoelectric point), and best match to NR protein database.

**Table 2:** Salivary gland transcripts of *Phlebotomus orientalis* – Melka Werer colony.

Cluster	Sequence name	Accession number	Proteome	MW	pI	Best match to NR protein database		
						Accession number	Species	E-value
PorMSP3	35.5 kDa salivary apyrase	KC170960	Y	35.63	8.83	ABB00906	*Phlebotomus perniciosus*	0.0
PorMSP4	35.2 kDa salivary apyrase	KC170961	Y	33.22	8.89	ADJ54077	*Phlebotomus tobbi*	0.0
PorMSP6	30 kDa antigen 5-related salivary protein	KC170962	Y	28.78	8.94	ABA43055	*Phlebotomus perniciosus*	1e-179
PorMSP8	28.8 kDa antigen 5-related salivary protein	KC170963	Y	28.78	8.94	ADJ54083	*Phlebotomus tobbi*	3e-151
PorMSP12	14.9 kDa PpSP15-like salivary protein	KC170964	Y	14.9	8.77	ADJ54084	*Phlebotomus tobbi*	3e-73
PorMSP15	24.53 kDa PpSP32-like salivary protein	KC170965		25.02	10.24	ADJ54102	*Phlebotomus tobbi*	1e-127
PorMSP23	42 kDa yellow-related salivary protein	KC170966	Y	41.59	6.09	ABA43049	*Phlebotomus perniciosus*	0.0
PorMSP24	42.6 kDa yellow-related salivary protein	KC170967	Y	42.31	8.07	ADJ54080	*Phlebotomus tobbi*	0.0
PorMSP27	putative alpha-amylase	KC170968		33.4	5.75	ACS93490	*Phlebotomus arabicus*	4e-178
PorMSP28	27.0 kDa D7-related salivary protein	KC170969	Y	27.27	7.53	ADJ54096	*Phlebotomus tobbi*	1e-156
PorMSP38	27.1 kDa D7-related salivary protein	KC170970	Y	26.94	8.26	ADJ54095	*Phlebotomus tobbi*	3e-162
PorMSP43	27 kDa D7-related salivary protein SP04B	KC170971		26.7	6.71	ABA43052	*Phlebotomus perniciosus*	1e-151
PorMSP65	38.8 kDa ParSP25-like salivary protein	KC170972		27.56	4.78	ADJ54098	*Phlebotomus tobbi*	6e-140
PorMSP67	27 kDa D7-related salivary protein	KC170973	Y	26.76	9.2	ABA43058	*Phlebotomus perniciosus*	6e-155
PorMSP74	13.8 kDa PpSP15-like salivary protein	KC170974	Y	13.92	9.18	ADJ54086	*Phlebotomus tobbi*	4e-70
PorMSP75	14.7 kDa PpSP15-like salivary protein	KC170975		14.7	7.99	ADJ54085	*Phlebotomus tobbi*	8e-62
PorMSP78	33 kDa salivary lufaxin	KC170976		18.78	8.4	ABA43054	*Phlebotomus perniciosus*	4e-99
PorMSP90	14.4 kDa PpSP15-like salivary protein	KC170977		14.32	8.73	ADJ54088	*Phlebotomus tobbi*	6e-77
PorMSP96	14.6 kDa PpSP15-like salivary protein	KC170978	Y	14.5	8.88	ADJ54089	*Phlebotomus tobbi*	1e-74
PorMSP101	41 kDa salivary endonuclease	KC170979		41.7	9.44	ABA43064	*Phlebotomus perniciosus*	0.0
PorMSP104	4.5 kDa-like salivary protein	KC170980		5.63	10.97	ADJ54097	*Phlebotomus tobbi*	3e-18
PorMSP108	salivary hyaluronidase	KC170981		35.6	7.98	ACS93505	*Phlebotomus arabicus*	2e-163
PorMSP129	30 kDa salivary phospholipase A2	KC170982		29.72	8.31	ABA43062	*Phlebotomus perniciosus*	0.0
PorMSP162	16 kDa salivary protein A	KC170983		15.97	5.04	ACS93506	*Phlebotomus arabicus*	1e-41
PorMSP169	3.7 kDa-like salivary protein	KC170984		3.93	9.16	ADJ54106	*Phlebotomus tobbi*	2e-7
PorMSP196	5.0 kDa-like salivary protein	KC170985		4.97	10.18	ADJ54105	*Phlebotomus tobbi*	2e-14

Putatively secreted salivary proteins from MW *Phlebotomus orientalis* colony with the number of cluster, GenBank accession number, presence in proteome, putative mature protein features (MW- molecular weight, pI- isoelectric point), and best match to NR protein database.

### Proteome analysis

**Figure 1:**
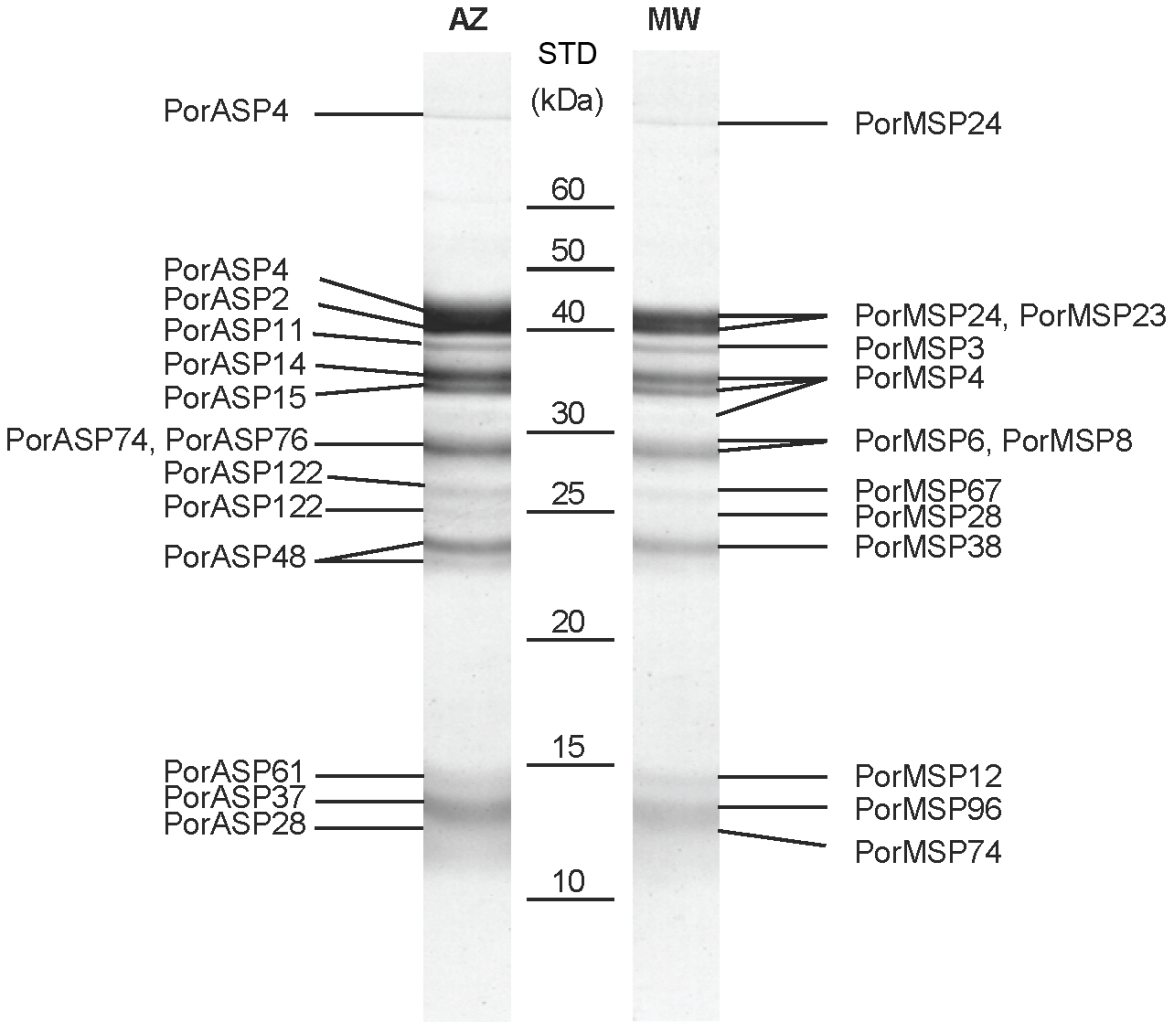
Proteomic analysis of salivary gland homogenates from *Phlebotomus orientalis*. *Phlebotomus orientalis* salivary proteins from Addis Zemen (AZ) and Melka Werer (MW) colonies (Ethiopia) were identified using Mass Spectrometry. The name of sequences contained in each protein band and molecular weight in kDa (STD/kDa) are indicated.

Salivary proteins presented in the proteome were identified by mass spectrometry and are shown in Figure 1. In both cDNA libraries, 12 salivary proteins were determined to be present in proteome. In Addis Zemen colony, the identified proteins were two yellow-related proteins (PorASP2/KC170933; PorASP4/KC170934), three apyrases (PorASP11/KC170935; PorASP14/KC170936; PorASP15/KC170937), two D7-related proteins (PorASP48/KC170943; PorASP122/KC170954), two antigen 5-related proteins (PorASP74/KC170947; PorASP76/KC170948), and three PpSP15-like proteins (PorASP28/KC170938; PorASP37/KC170940; PorASP61/KC170944) (Figure 1). In Melka Werer colony, the identified proteins were two yellow-related proteins (PorMSP23/KC170966; PorMSP24/KC170967), two apyrases (PorMSP3/KC170960; PorMSP4/KC170961), three D7-related proteins (PorMSP28/KC170969; PorMSP38/KC170970; PorMSP67/KC170973), two antigen 5-related proteins (PorMSP6/KC170962; PorMSP8/KC170963), and three PpSP15-like proteins (PorMSP12/KC170964; PorMSP74/KC170974; PorMSP96/KC170978) (Figure 1). Except for apyrase, none of the salivary enzymes identified in *P. orientalis* transcriptomes were detected in proteome analysis, even though all of the nucletoide sequences coding for these salivary proteins possessed signal peptides. It might be explained by the fact that extremely active enzymes do not need a huge amount of protein to be effective.

### Yellow-related proteins

**Figure 2:**
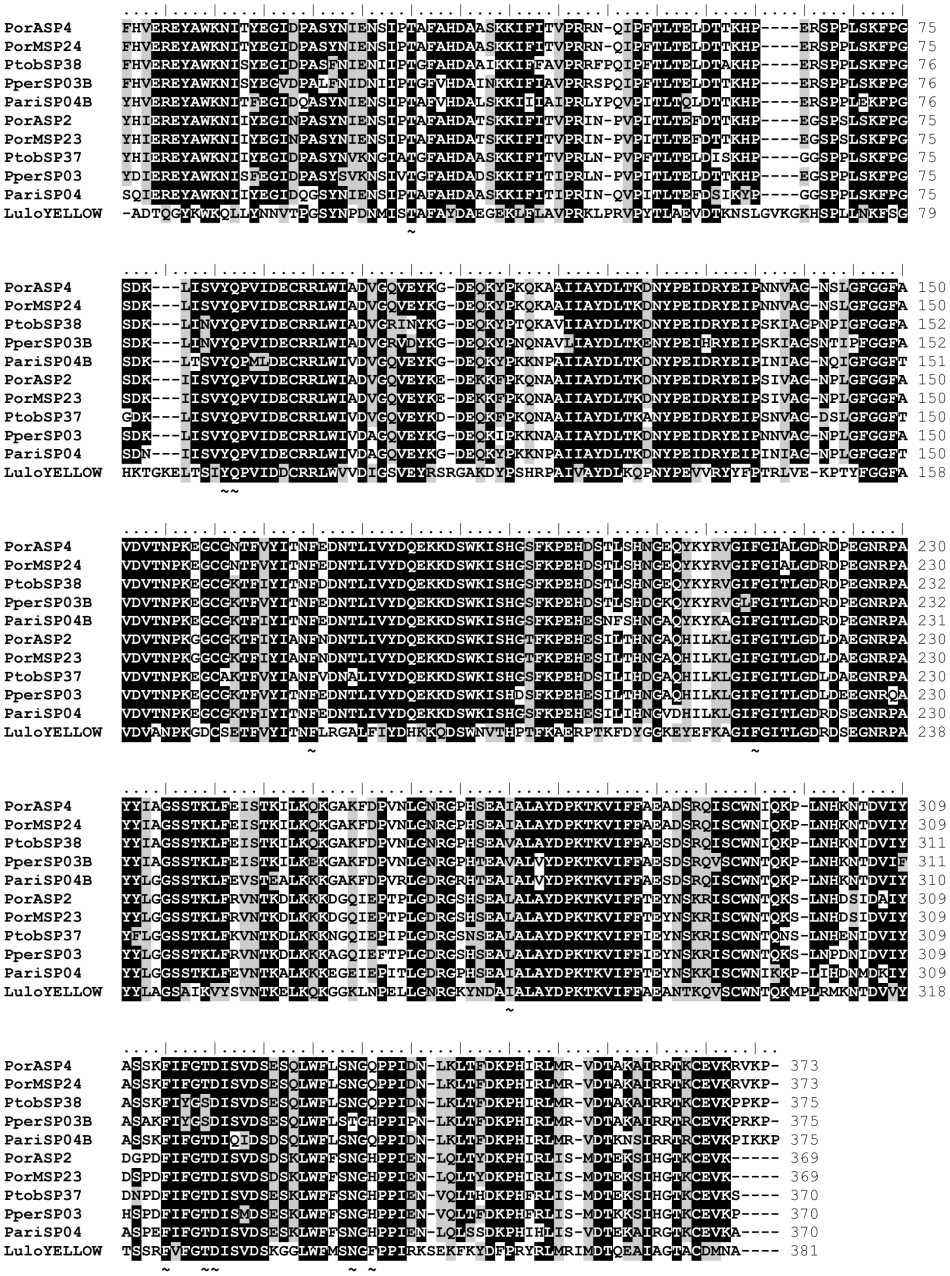
Multiple sequence alignment of the sand fly yellow-related protein family. Multiple sequence alignment of yellow-related salivary proteins from *Phlebotomus ariasi* (Pari), *Phlebotomus perniciosus* (Pper), *Phlebotomus orientalis* Addis Zemen colony (PorA), *P. orientalis* Melka Werer colony (PorM), *Phlebotomus tobbi* (Ptob), and *Lutzomyia longipalpis* (Lulo). Sequence names and the number of amino acids per line are indicated. Identical amino acid residues are highlighted black and similar residues grey. Specific symbols indicate: **∼** amino acid motif binding prohemostatic and proinflammatory biogenic amines. The symbols refer to the lines above.

Yellow-related proteins are abundantly expressed in the sand fly salivary glands and have been detected in the saliva of all sand fly species tested, to date [7], [26]–[28], [31], [50], [52]–[56]. Two yellow-related proteins were found in the cDNA library of the AZ (PorASP2/KC170933; PorASP4/KC170934) as well as the MW (PorMSP23/KC170966; PorMSP24/KC170967) *P. orientalis* colony). All four *P. orientalis* yellow-related proteins had similar predicted molecular mass (41.5–42.3 kDa) and wide range of pI (6.1–8.1) (Table 1, Table 2). All obtained sequences contained the entire major royal jelly protein (MRJP) domain, which is characteristic for the yellow-related proteins. Some advances have been also made in describing the function of sand fly yellow-related proteins. It was shown that recombinant yellow-related proteins from *L. longipalpis* saliva (AAD32198, AAS05318) act as high affinity binders of prohemostatic and proinflammatory biogenic amines such as serotonin, catecholamines and histamine [12]. Similarly, the amino acid motif present in the ligand binding pocket of *L. longipalpis* (T-x(52,63)-Y-Q-x(85,90)-[FY]-x(44,46)-F-x(54)-[IVL]-x(45,46)-[FY]-x-[TS]-D-x(13)-[NT]-x-[QHFL]) was discovered in the yellow-related proteins of *L. ayacuchensis* (BAM69111, BAM69185, BAM69109, BAM69110) [56] and *L. intermedia* (AFP99235) [52], but also in *P. orientalis* and other sand fly species from the subgenus *Larroussius* tested (Figure 2). These findings suggest similar anti-inflammatory function of these salivary proteins in other *Lutzomyia* and *Phlebotomus* sand fly species [12] and could potentially explain the lectin-like properties of 42 kDa yellow-related protein from *P. duboscqi* saliva [57]. Sand fly yellow-related proteins share homology with the yellow protein of *Drosophila melanogaster* and to the MRJPs of honeybees. Similarly, sequences with homology to *D. melanogaster* yellow protein were also found in other bloodsucking insects; for example, the mosquito *Aedes aegypti* [58] and the tsetse fly *Glossina morsitans morsitans* [59].

**Figure 3:**
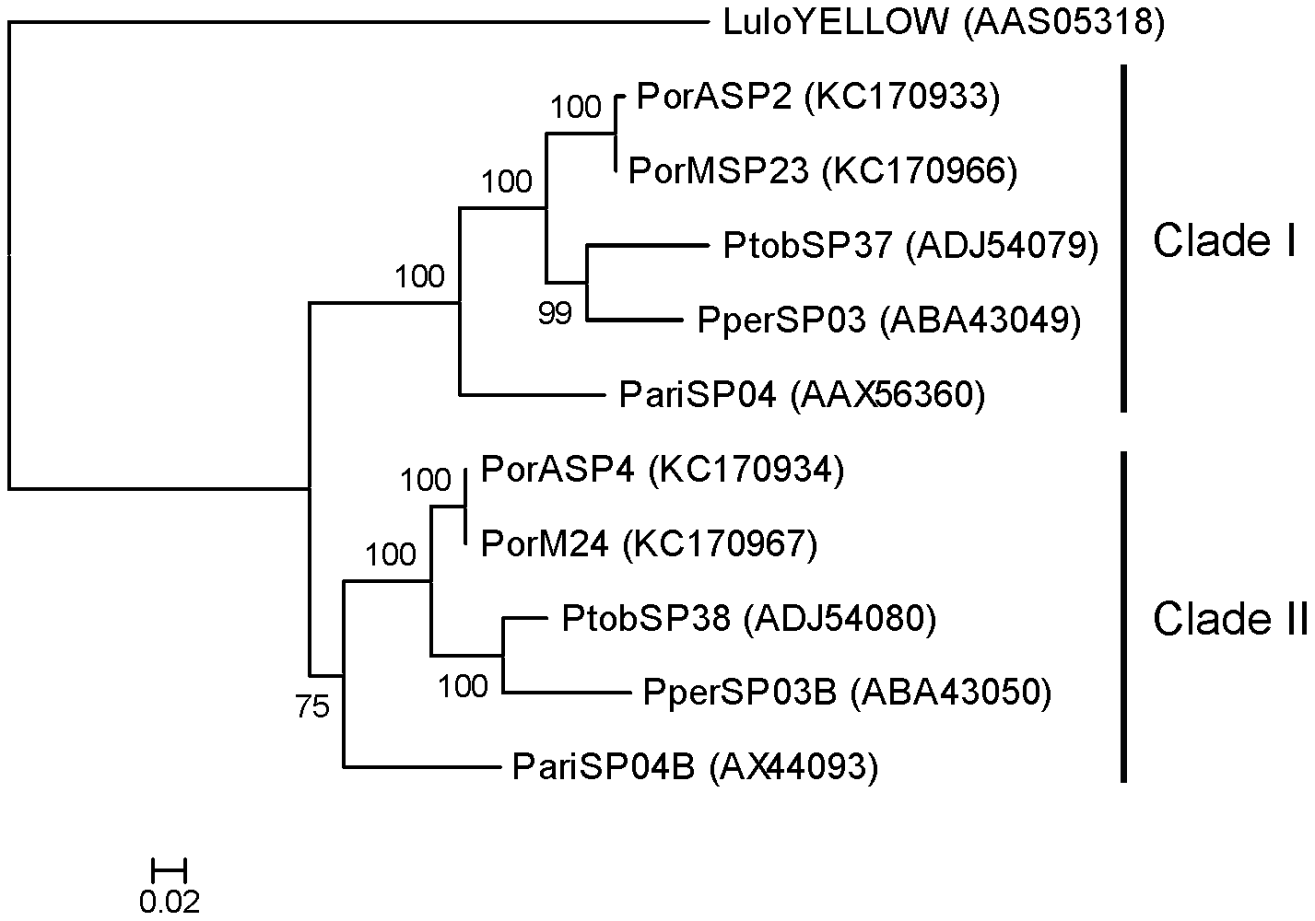
Phylogenetic analysis of the yellow-related family of sand fly salivary proteins. Phylogenetic analysis of yellow-related salivary proteins from *Phlebotomus ariasi* (Pari), *Phlebotomus perniciosus* (Pper), *Phlebotomus orientalis* Addis Zemen colony (PorA), *P. orientalis* Melka Werer colony (PorM), *Phlebotomus tobbi* (Ptob), and *Lutzomyia longipalpis* (Lulo). The JTT model was used for this phylogenic analysis. Sequence names, GenBank accession numbers and branch values are indicated. Yellow-related salivary proteins from *Larroussius* sand fly species are divided into two distinct clades (Clade I, II).

Phylogenetic analysis shows that yellow-related proteins from *P. orientalis* saliva are divided into two clades (Figure 3). Both clades are represented by two yellow-related salivary proteins, one from each *P. orientalis* cDNA library (clade I - PorASP2, PorMSP23; clade II - PorASP4, PorMSP24). *Phlebotomus orientalis* sequences within the same clade revealed high degree of identity (99 and 100%, respectively), while comparison between clades showed 77% identity (Figure 2). Yellow-related proteins of other sand fly species from subgenus *Larroussius* were also split into two clades and these sequences are closely related to *P. orientalis* proteins (83–91% identity) (Figures 2 and 3).

Yellow-related proteins were shown to be highly immunogenic. These proteins were recognized by sera of repeatedly bitten hosts such as mice [5], [15], [60], hamsters [60], dogs [4], [61]–[63], foxes [4], [64], and humans [4], [15], [65]–[67]. Furthermore, recombinant yellow-related salivary proteins (AAD32198, AAS05318) were successfully employed as the markers of sand fly exposure for individuals in endemic areas [3], [4]. Importantly, *L. longipalpis* salivary yellow-related proteins seem to be promising candidates for anti-*Leishmania* vaccine. Inoculation of plasmids coding for *L. longipalpis* yellow-related salivary proteins (AAD32198, AAS05318) into the skin elicited a strong delayed type hypersensitivity (DTH) reaction in various hosts [8], [12], [68], which resulted in efficient killing of *Le. infantum chagasi* parasites *in vitro* [68] and protection against *Le. major* infection *in vivo* [12], [13].

According to the glycosylation prediction servers (NetNGlyc and NetOGlyc) we found out that PorASP4 and PorMSP24 are likely N-glycosylated and have three threonine sites for potential O-glycosylation. PorASP2 and PorMSP23 have four threonines for potential O-glycosylation and no N-glycosylation was predicted.

### Apyrase

Sequences coding for apyrase were detected in the cDNA libraries of both the AZ (PorASP11/KC170935; PorASP14/KC170936; PorASP15/KC170937) and the MW (PorMSP3/KC170960; PorMSP4/KC170961) *P. orientalis* colonies. All sequences had similar predicted molecular mass (33.2–35.6 kDa) and pI ranged from 8.8 to 10.0. Apyrase is the principal anti-platelet aggregation enzyme which hydrolyses ATP and ADP to AMP and orthophosphate, thereby blocks the physiological signal of damaged cells and tissues. This enzyme has been found in the saliva of all sand fly species tested, to date [7], [26]–[28], [31], [50], [52]–[56], but also in the saliva of other medically important hematophagous insect such as triatomine bugs (e.g. [69]), mosquitoes (e.g. [70]), fleas (e.g. [71]), tsetse flies (e.g. [72]), biting midges (e.g. [73]), and horseflies (e.g. [74]). Interestingly, apyrase has also been described in non-bloodsucking insects; for example, *Helicoverpa zea* [75] and *D. melanogaster* [76], indicating that apyrase may have a broader functional potential than only the facilitation of blood acquisition.

**Figure 4:**
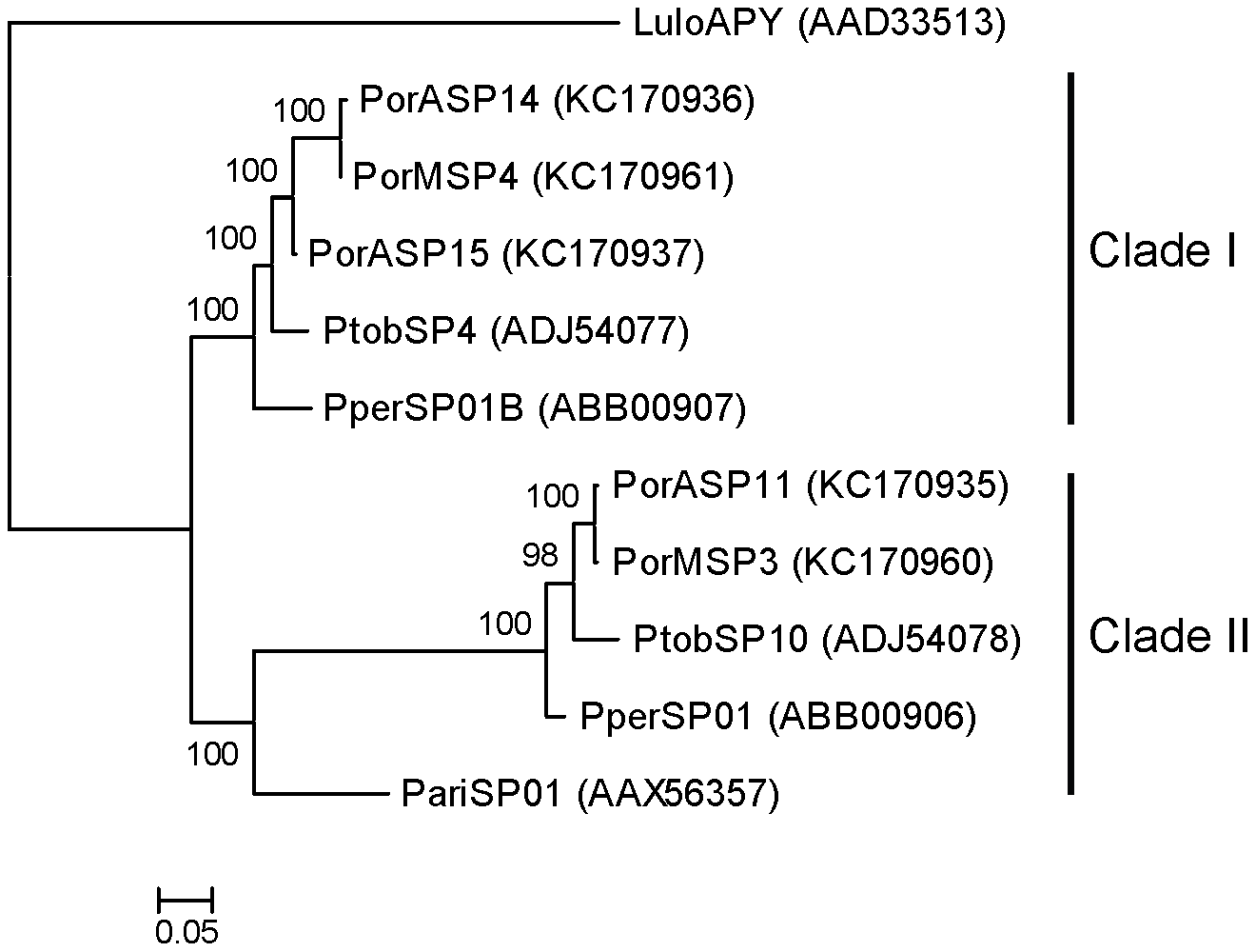
Phylogenetic analysis of the apyrase family of sand fly salivary proteins. Phylogenetic analysis of salivary apyrases from *Phlebotomus ariasi* (Pari), *Phlebotomus perniciosus* (Pper), *Phlebotomus orientalis* Addis Zemen colony (PorA), *P. orientalis* Melka Werer colony (PorM), *Phlebotomus tobbi* (Ptob), and *Lutzomyia longipalpis* (Lulo). The WAG model was used for this phylogenic analysis. Sequence names, GenBank accession numbers and branch values are indicated. Apyrases from *Larroussius* sand fly species are divided into two distinct clades (Clade I, II).

Apyrases of the bloodfeeding insect are divided into three families: GTPase/CD-39, 5′- nucleotidase, and *Cimex* type (reviewed in [77]). Apyrases from *P. orientalis* colonies, as well as from other sand fly species, are homologous to the bed bug apyrase which defined the *Cimex* type family [78]. Phylogenic analysis showed that salivary apyrases from *P. orientalis* colonies are separated into two clades (Figure 4). Clade I includes two apyrases from the AZ colony and one from the MW colony (PorASP14, PorASP15, and PorMSP4), as the analogue of the second apyrase in MW colony was excluded from the phylogenetic analysis due to absence of signal peptide and the low quality of sequence. Clade II contains two apyrases, one from each colony (PorASP11 and PorMSP3). Sequences of *P. orientalis* apyrase within the same clade revealed high degree of identity (95–99%), whereas the comparison between the clades showed an identity of 66% (Figure S1). Comparison with other sand fly species from the subgenus *Larroussius* showed that apyrases from *P. tobbi* (ADJ54077, ADJ54078) and *P. perniciosus* (ABB00906, ABB00907) saliva are closely related to *P. orientalis*, while apyrase from *P. ariasi* (AAX56357) saliva is more distinct (Figures 4 and S1).

Apyrase activity has been demonstrated in the saliva of *L. longipalpis* [53], *P. papatasi* [48], [79], *P. duboscqi* [80], *P. perniciosus*, *P. argentipes*, and *P. colabaensis* [48]. In our experiments, apyrase activity was measured in the saliva of both AZ and MW *P. orientalis* colonies (Table 3). ATPase as well as ADPase activity, determined per the pair of salivary glands, was slightly higher in AZ colony, but recalculation of enzymatic activity per milligram of total proteins showed that apyrase activity in both colonies is comparable. Also, the ATPase/ADPase ratio was equal in both colonies (Table 3). Comparison of *P. orientalis* apyrase activity with *P. perniciosus* [48] revealed that ATPase and ADPase activities determined per pair of glands are comparable. Additionally, in accordance with previous data [48], [53], [79]–[81], we showed that *P. orientalis* apyrase activity is dependent on presence of Ca^2+^ but not on Mg^2+^ ions.

**Table 3:** Salivary apyrase in two *P. orientalis* colonies originated from Melka Werer (MW) and Addis Zemen (AZ), Ethiopia.

		*P. orientalis* AZ	*P. orientalis* MW	*P. papatasi*
Total protein in µg/gland pair		0.61±0.05	0.52±0.06	0.68±0.07
Mean specific apyrase activity* at 37°C, pH 8.5:				
mUnits/pair of glands **	ATPase	87.9±2.9	74.3±3.9	77.9±5.3
	ADPase	99.3±6.7	84.2±6.7	89.80±6.9
Units/mg of total protein	ATPase	144	143	115
	ADPase	163	162	132
ATPase/ADPase ratio		0.88	0.88	0,87
pH optimum		8.5	8.5	nd
Activator cation		Ca^2+^	Ca^2+^	Ca^2+^

*Phlebotomus papatasi* was used as the control.

*One unit of enzyme activity is defined as the amount of enzyme that releases one micromole of orthophosphate per minute from the nucleotide substrate at specified assay conditions.

**Individual specific activity was calculated per gland pair as *P. papatasi* is characterized by dissimilar size of salivary glands [45].

Besides the anti-hemostatic effect of this enzyme, apyrase is also known as a powerful antigen. Specific antibodies from dogs bitten by *P. perniciosus* in the field, as well as under laboratory conditions, reacted strongly with two salivary apyrases [63]. Apyrases from *P. perniciosus*, *P. papatasi*, and *P. argentipes* saliva were also recognized by sera of mice and hamsters immunized by homologous antigen [5], [60], [82]. Furthermore, bacterially expressed recombinant *P. duboscqi* apyrase (ABI20147) was also recognized by specific antibodies from mice immunized with *P. duboscqi* saliva [80], suggesting that antibody recognition is not solely targeted to the glycosylated parts of the antigen. On the other hand, inoculation of bacterially expressed recombinant *L. longipalpis* apyrase (AAD33513) into C57BL/6 mice did not elicit either antibody response or DTH reaction [12]. These data indicates that the immunogenicity of the protein or saccharidic part of antigen may vary in different sand fly species. According to the glycosylation prediction servers (NetNGlyc and NetOGlyc), *P. orientalis* apyrases PorASP14, PorASP15, and PorMSP4 are N-glycosylated, while no O-glycosylation sites were predicted.

### Hyaluronidase

Hyaluronidase is an enzyme that degrades hyaluronic acid and other glycosaminoglycan constituents abundantly present in the vertebrate extracellular matrix. It is a well-known allergen occurring in the venom of bees, hornets, wasps, spiders, and snakes (reviewed in [83], [84]), but hyaluronidase activity was also observed in the saliva of various bloodsucking Diptera [28], [31], [45], [85], [86]. Previously published data showed that hyaluronidase is able to promote the spreading of other components of bloodfeeding insect saliva within the skin, as well as to enhance the success of potential parasite transmission [86]. Although positive enzymatic activity was detected in all sand fly species tested to date [28], [31], [45], [53], [85], [86], transcripts coding for putative hyaluronidase were ascertained only in four of them, namely *P. arabicus* (ACS93505), *P. tobbi* (AEK98519), *L. longipalpis* (AAD32195), *L. intermedia* (AFP99265) [28], [31], [52]–[54], and in both *P. orientalis* colonies (PorASP112/KC170958; PorMSP108/KC170981) (Table 1, Table 2). The predicted molecular mass of AZ and MW hyaluronidase was 37.2 and 35.6 kDa, respectively, and the pI was 6.5 and 8.0, respectively.

Hyaluronidase activity measured in the *P. orientalis* saliva was found to be lower than the activity of other *Larroussius* species tested by [28]. While hyaluronidase activity expressed in the relative Turbidity Reducing Units (rRTU) reached approximately 0.62 rTRU/gland in *P. tobbi* and 0.48 rTRU/gland in *P. perniciosus* [28], enzymatic activity in *P. orientalis* saliva was 0.22 rTRU per gland (0.22±0.036 rTRU in AZ and 0.215±0.045 rTRU in MW).

**Figure 5:**
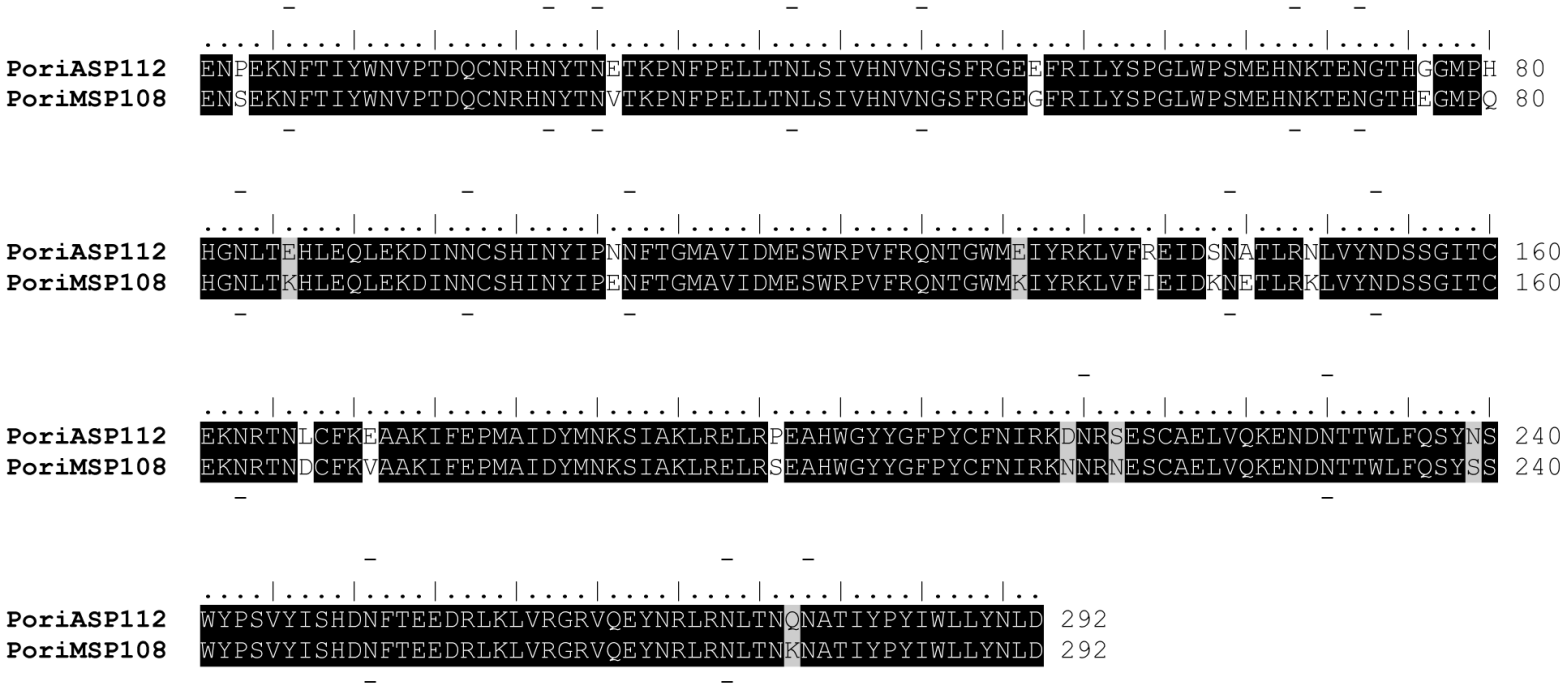
Sequence alignment of the *P. orientalis* hyaluronidase. Sequence alignment of salivary hyaluronidase from *Phlebotomus orientalis* Addis Zemen (PorA) and *Phlebotomus orientalis* Melka Werer (PorM) colonies represented by hyaluronidase protein domains. Sequence names and the number of amino acids per line are indicated. Identical amino acid residues are highlighted black and similar residues grey. Specific symbols indicate: - N-glycosylation prediction sites. The symbols above and under the lines refer to the Addis Zemen and Melka Werer sequences, respectively.

*Phlebotomus orientalis* salivary hyaluronidase of AZ and MW colonies revealed identity reaching 94% (Figure 5). High degree of identity was achieved with *P. tobbi* sequences (AEK98519) (89–93%), followed by *P. arabicus* (ACS93505) (80–83%), *L. longipalpis* (AAD32195) (56–58%), and *L. intermedia* (AFP99265) (47–48%).

Moreover, glycosylation prediction servers (NetNGlyc and NetOGlyc) showed that salivary hyaluronidase is the most glycosylated protein in both colonies, with seventeen predicted N-glycosylation sites in the AZ and sixteen in the MW colony (Figure 5).

### Other enzymes

**Figure 6:**
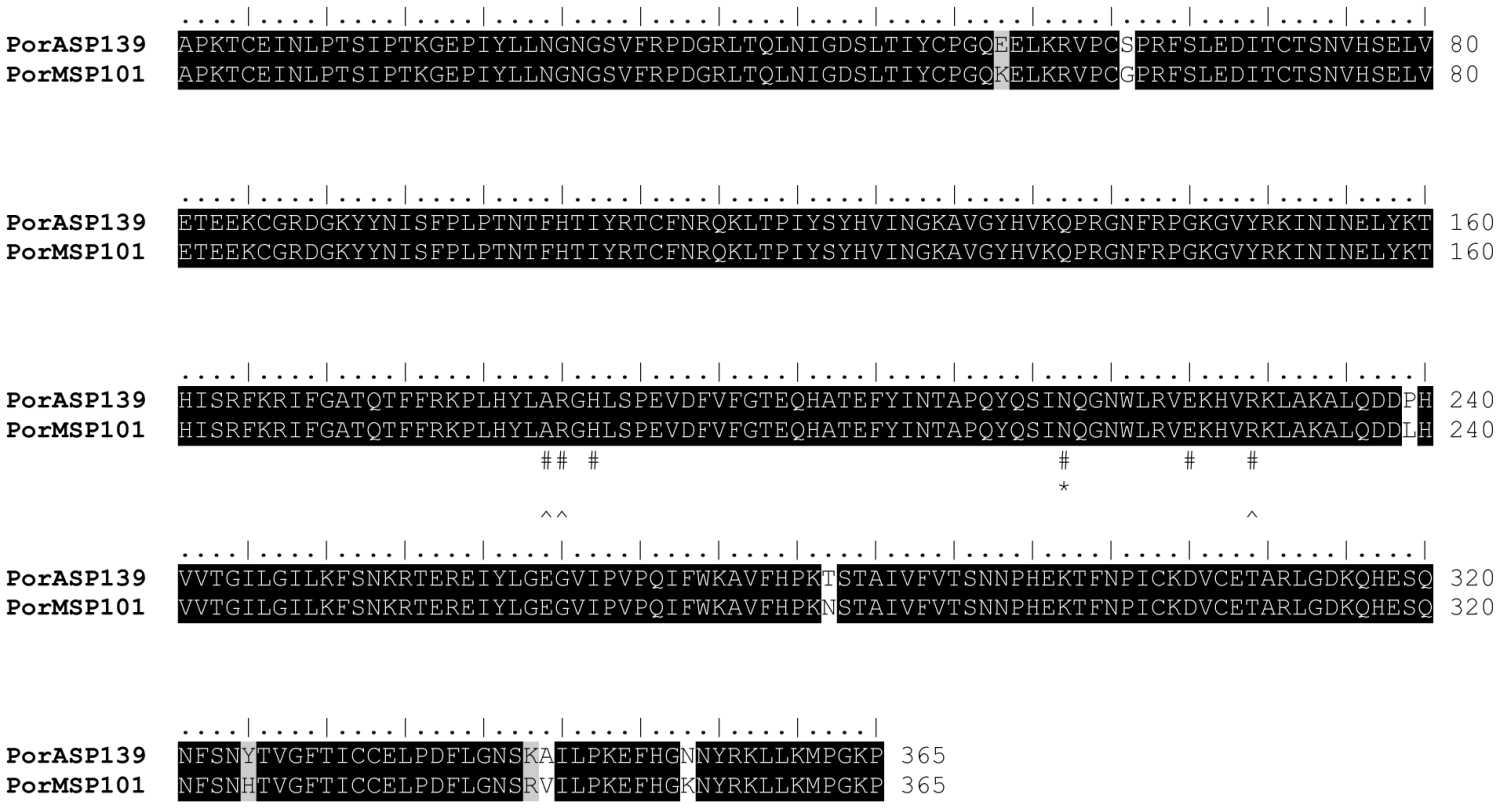
Sequence alignment of the *P. orientalis* endonuclease. Sequence alignment of endonucleases from *Phlebotomus orientalis* Addis Zemen (PorA) and *Phlebotomus orientalis* Melka Werer (PorM) colonies. Sequence names and the number of amino acids per line are indicated. Identical amino acid residues are highlighted black and similar residues grey. Specific symbols indicate: # enzyme active sites, * Mg^2+^ binding site, and ∧ substrate binding sites. The specific symbol refers to the sequence line above.

Another enzyme that was identified among the transcripts from both *P. orientalis* cDNA libraries is a putative endonuclease (PorASP139/KC170955; PorMSP101/KC170979) (Table 1, Table 2). Addis Zemen, as well as Melka Werer, sequences contained the NUC Smart motif, which is typical for DNA/RNA non-specific endonucleases and phosphodiesterases. Predicted molecular mass of both AZ and MW endonucleases was 41.7 kDa and predicted pI was 9.3 and 9.4, respectively. Endonuclease function in sand fly saliva is still unclear; however, properties that facilitate blood acquisition are assumed. Endonucleases were detected in salivary gland cDNA libraries of some sand flies species tested [26], [27], [31], [52], [54]–[56], but also in another bloodsucking Diptera [87], [88]. Sequences of AZ and MW *P. orientalis* colony coding for endonuclease revealed 97% identity and furthermore, there was no difference in the numbers and positions of the active sites, Mg^2+^ binding sites, and substrate binding sites (Figure 6). *Phlebotomus perniciosus* salivary endonuclease (ABA43064) was found to be the most relative sequence (92% identity), while homology of *P. orientalis* enzymes with other sand fly endonucleases ranged between 44–80%. Endonuclease was also shown to have antigenic properties; sera of dogs from an endemic area of VL in Italy, as well as dogs experimentally bitten by *P. perniciosus*, reacted with a 41 kDa salivary protein identified as the endonuclease (ABA43064) [63].

**Figure 7:**
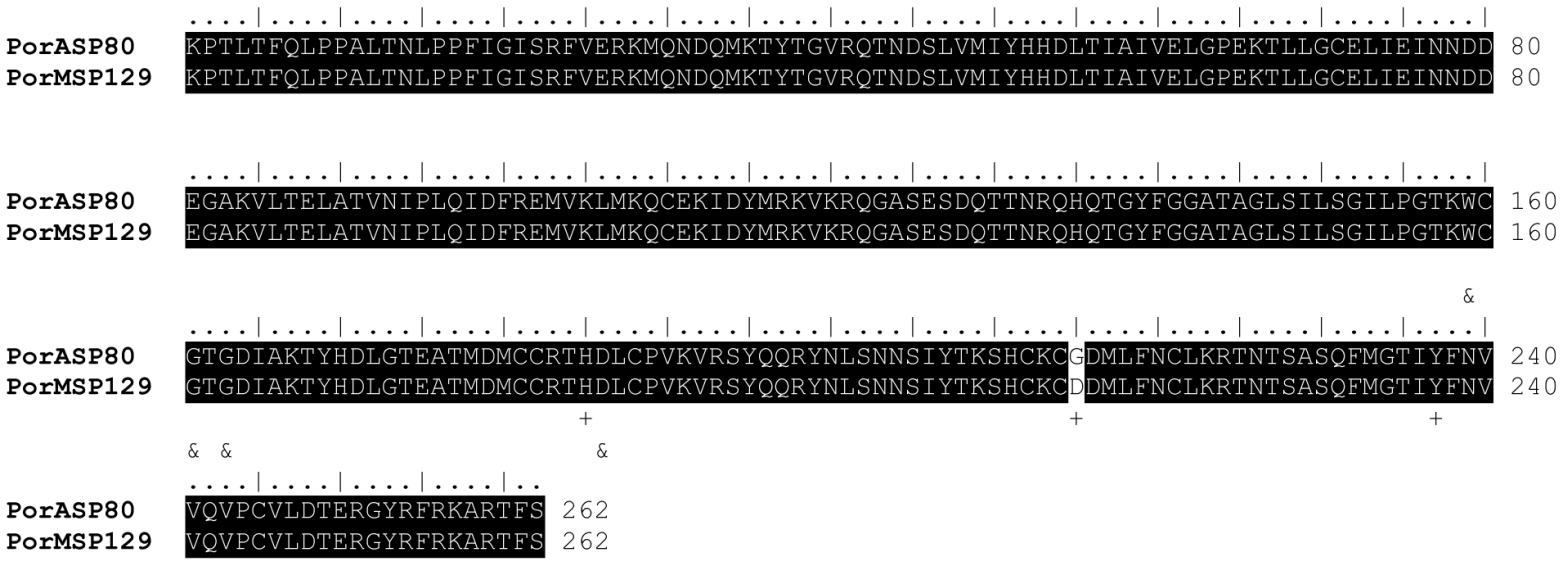
Sequence alignment of the *P. orientalis* phospholipase. Sequence alignment of phospholipases from *Phlebotomus orientalis* Addis Zemen (PorA) and *Phlebotomus orientalis* Melka Werer (PorM) colonies. Sequence names and the number of amino acids per line are indicated. Identical amino acid residues are highlighted black. Specific symbols indicate: + catalytic sites, & metal binding sites. The specific symbol refers to the sequence line above.

Transcripts coding for a putative phospholipase A2 (PLA2) were detected in both *P. orientalis* cDNA libraries (PorASP80/KC170949; PorMSP129/KC170982) (Table 1, Table 2). In AZ and MW sequences, the whole PLA2 domain was present. The predicted molecular mass of PLA2 was 29.7 kDa and pI was 8.4 and 8.3 for AZ and MW, respectively. PLA2 was described as the main allergen in the hymenopteran venom (reviewed in [84]), however, its allergenic effect in sand flies remains to be elucidated. Sequences coding for PLA2 revealed a high degree of conservancy between AZ and MW colonies as well as among various sand fly species. The AZ and MW *P. orientalis* PLA2 were almost identical (99%) and the metal binding sites were present on the same positions (Figure 7). Similarly, the catalytic sites were detected on the same positions and amino acids in both colonies, with the exception of the catalytic site on 215^th^ amino acid, where glycine present in AZ colony was in MW replaced by aspartic acid (Figure 7). Within the *Larroussius* subgenus, the homology of *P. orientalis* PLA2 reached 99% with *P. perniciosus* (ABA43062) and 94% with *P. ariasi* (AAX54852). Moreover, comparing the PLA2 enzymes of *P. orientalis* and *P. arabicus* (ACS93491), subgenus *Adlerius*, showed 88% identity.

A single 3′truncated transcript coding for a putative α-amylase was detected in the salivary gland cDNA library of MW *P. orientalis* colony (PorMSP27/KC170968) (Table 2), but no homologous sequences were found in the AZ colony. Amylase is an enzyme which is likely not involved in the bloodfeeding process, but participates in dietary sugar digestion (reviewed in [89]). Transcripts coding for α-amylase were detected in the salivary gland cDNA libraries of *L. longipalpis* (AAD32192) [53], *P. arabicus* (ACS93490) [31], and in *P. papatasi* (AAD32192) [55]. Alpha-amylase activity was detected in the sand fly salivary glands [53], [90], [91]. The predicted molecular weight of the *P. orientalis* MW amylase was 33.4 kDa and the predicted pI was, 5.8 (Table 2). Amino acid sequence alignment of *P. orientalis* α-amylase shows an 88% identity with the *P. arabicus* α-amylase (ACS93490) and 82% identity with *L. longipalpis* α-amylase (AAD32192).

A single sequence containing signal peptide, truncated in the 3′region (missing stop codon), coding for a putative salivary pyrophosphatase (PorASP262/KC170959) was ascertained in the AZ cDNA library (Table 1). The predicted molecular mass was 32.9 kDa and the predicted pI was 7.2. Pyrophosphatase was also detected in the MW colony, but these sequences did not contain signal peptides. Nonetheless, the identity of AZ and MW pyrophosphatases reached 99%. Salivary pyrophosphatase was found also in saliva of other sand fly species from the genus *Phlebotomus* such as *P. duboscqi* (ABI20154) [50], *P. argentipes* (ABA12155) [26], and *P. arabicus* (ACS93498) [31]. Transcripts coding for pyrophosphatase did not reveal a high degree of conservancy, as homology of AZ or MW *P. orientalis* enzymes with the aforementioned sequences ranged between 39–74%.

### D7-related proteins

D7-related proteins belong to the odorant-binding protein superfamily, which is composed of pheromone-binding proteins (PBP) and general-odorant-binding proteins (GOBP). D7 proteins are commonly present in the salivary glands of various bloodfeeding insect such as mosquitoes (e.g. [70]), black flies (e.g. [92]), biting midges (e.g. [93]) and sand flies [7], [26]–[28], [31], [50], [52], [54]–[56], [94]. Moreover, proteins belonging to the insect odorant binding protein family were recently detected in *L. longipalpis* pheromone glands [95].

In the *P. orientalis* cDNA libraries we found three different D7-related proteins in the AZ colony (PorASP46/KC170942, PorASP48/KC170943, PorASP122/KC170954) and four in the MW colony (PorMSP28/KC170969, PorMSP38/KC170970, PorMSP43/KC170971, PorMSP67/KC170973) (Table 1, Table 2). They all had a similar predicted molecular mass (26.7–27.3 kDa) and wide range of pI (6.4–9.2).

The function of sand fly salivary D7-related proteins remains unknown, although it might be similar to mosquito D7 proteins; either as a binder of biogenic amines and/or eicosanoids [96], [97] or as an anticoagulant [98], [99].

**Figure 8:**
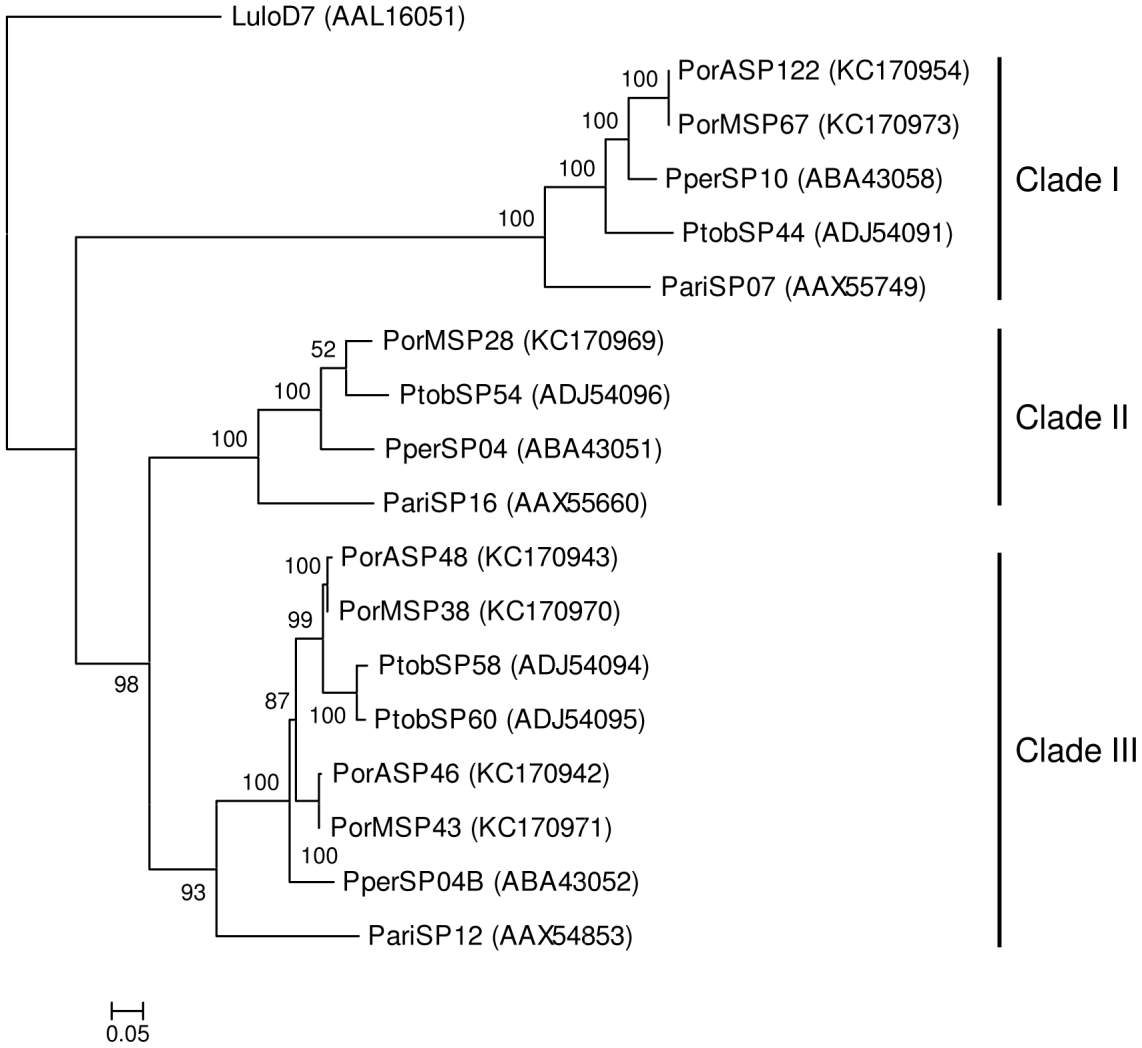
Phylogenetic analysis of the D7-related family of sand fly salivary proteins. Phylogenetic analysis of D7-related salivary proteins from *Phlebotomus ariasi* (Pari), *Phlebotomus perniciosus* (Pper), *Phlebotomus orientalis* Addis Zemen colony (PorA), *P. orientalis* Melka Werer colony (PorM), *Phlebotomus tobbi* (Ptob), and *Lutzomyia longipalpis* (Lulo). The WAG model was used for this phylogenic analysis. Sequence names, GenBank accession numbers and branch values are indicated. D7-related proteins from *Larroussius* sand fly species are divided into three distinct clades (Clade I–III).

Phylogenetic analysis showed that *P. orientalis* D7-related salivary proteins are divided into three clades (Figure 8). Clade I contains two 100% identical *P. orientalis* D7-related proteins, one from each cDNA library (PorASP122, PorMSP67). Clade II contains only one *P. orientalis* protein from the MW colony (PorMSP28), as the analogue from AZ colony did not contain the signal peptide sequence and therefore was not included into the phylogenetic analysis. Clade III includes two proteins from each library, PorASP46 and PorASP48 from the AZ colony and PorMSP38 and PorMSP43 from the MW colony. *Phlebotomus orientalis* D7-related proteins within clade III form two distinct subclades (PorASP48, PorMSP38 and PorASP46, PorMSP43), where the identity reached 99%. Overall, the identity in the clade III was 92%. Sequences coding for salivary D7-related proteins in *P. orientalis* species did not reveal high degree of conservancy as the alignment of all D7-related proteins from both colonies reached only 31% identity (Figure S2). Comparison with other sand fly species from subgenus *Larroussius* showed that *P. orientalis* D7-related proteins are more related to *P. tobbi* and *P. perniciosus* than to *P. ariasi* (Figures 8 and S2).

Based on the glycosylation prediction servers (NetNGlyc and NetOGlyc), we found out that D7-related proteins have very limited glycosylation sites. Only those D7-related proteins occurring within clade I (PorASP122 and PorMSP67) were predicted to have N-glycosylation, while the others are likely not glycosylated at all. Similarly, only those D7-related proteins of *P. perniciosus*, *P. tobbi*, and *P. ariasi* included in the clade I are likely N-glycosylated, moreover, the glycosylation sites are predicted in all *Larroussius* sand fly species on the same positions. These data strengthens the idea that the proteins from different clades might have different molecular functions even though they are all D7-related proteins. Furthermore, mixtures of glycosylated and non-glycosylated D7-related proteins were previously detected in other sand fly species such as *P. arabicus* or *P. tobbi* [28], [31].

D7-related proteins are highly antigenic and were recognized by specific antibodies from the sera of repeatedly bitten hosts, regardless of natural [15], [61], [63], [67] or experimental exposure [5], [60], [62], [63], [82]. Recombinant *P. ariasi* D7-related protein (AAX55749) elicited the production of specific humoral response in immunized mice [27]. Anti-*P. papatasi* saliva antibodies reacted with the 30 kDa recombinant *P. papatasi* D7-related protein (AAL11049) [5], but the same protein was not recognized by the human sera from an endemic area of CL in Tunisia [100]. Moreover, recombinant 28 kDa D7-related protein from *P. papatasi* saliva (AAL11048) was not targeted by the specific antibodies of immunized mice [5]. Thus, a broad use of D7-related salivary proteins as the reliable marker of sand fly exposure is not likely. Importantly, no significant cellular immunity was observed in various hosts after the inoculation of DNA plasmids coding for D7-related sand fly salivary proteins [12], [27], [68].

### PpSP15-like proteins

Transcripts coding for PpSP15-like proteins represented the most abundant family in *P. orientalis* cDNA libraries. PpSP15-like proteins were detected in both the AZ (PorASP28/KC170938; PorASP31/KC170939; PorASP37/KC170940; PorASP61/KC170944; PorASP64/KC170945) and the MW colony (PorMSP12/KC170964; PorMSP74/KC170974; PorMSP75/KC170975; PorMSP90/KC170977; PorMSP96/KC170978) (Table 1, Table 2). The predicted molecular mass ranged from 13.9 to 14.9 and the isoelectric point was slightly basic (8.0–9.2).

**Figure 9:**
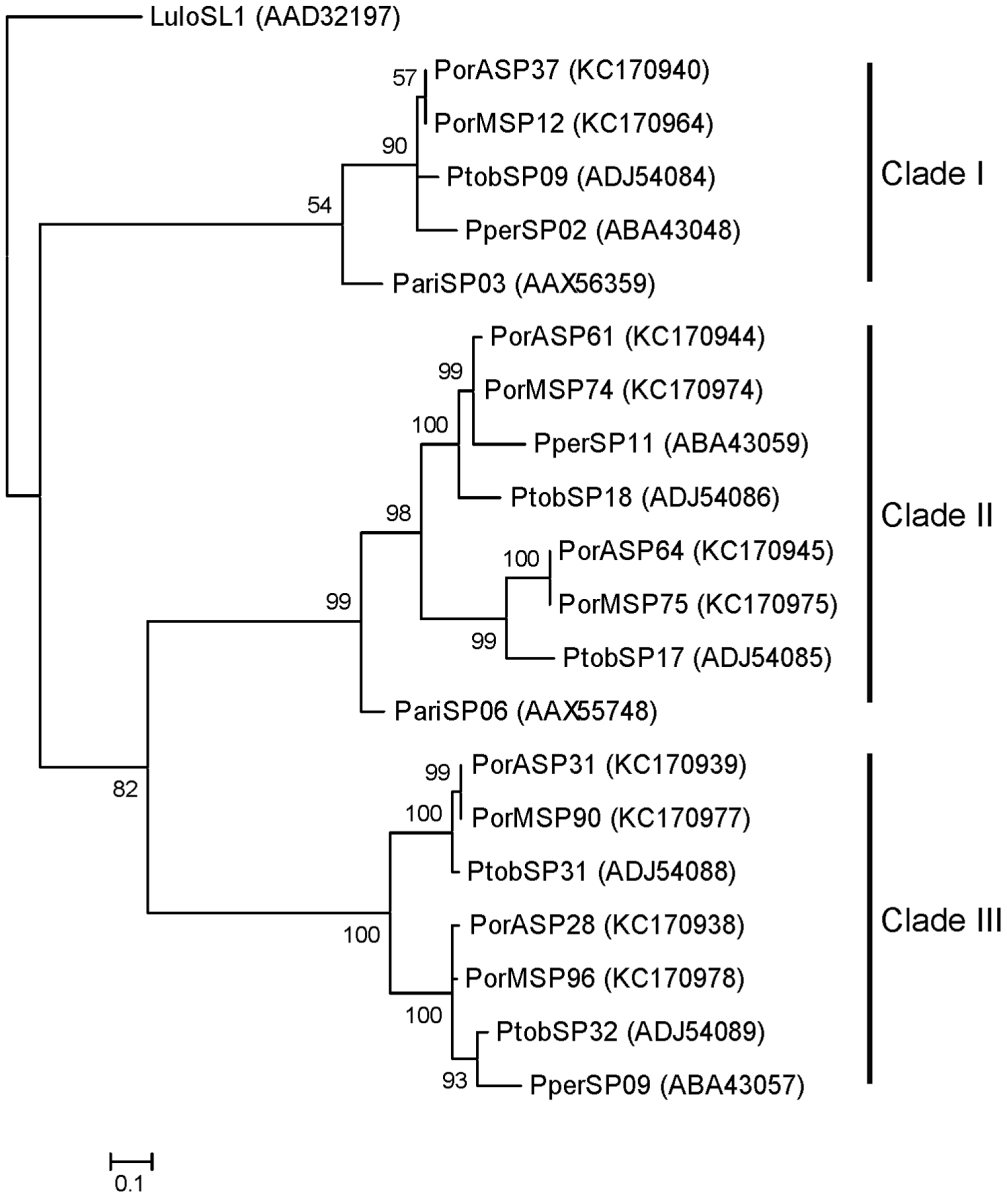
Phylogenetic analysis of the PpSP15-like family of sand fly salivary proteins. Phylogenetic analysis of PpSP15-like salivary proteins from *Phlebotomus ariasi* (Pari), *Phlebotomus perniciosus* (Pper), *Phlebotomus orientalis* Addis Zemen colony (PorA), *P. orientalis* Melka Werer colony (PorM), *Phlebotomus tobbi* (Ptob), and *Lutzomyia longipalpis* (Lulo). The JTT model was used for this phylogenic analysis. Sequence names, GenBank accession numbers and branch values are indicated. PpSP15-like proteins from *Larroussius* sand fly species are divided into three distinct clades (Clade I–III).

Phylogenetic analysis showed that *P. orientalis* PpSP15-like proteins are divided into three clades. Clade I contains two *P. orientalis* PpSP15-like proteins, one from each library (PorASP37, PorMSP12), which have an identity of 100%. Clades II and III each contain four *P. orientalis* proteins, two from each library (clade II: PorASP61, PorASP64, PorMSP74, PorMSP75; clade III: PorASP28, PorASP31, PorMSP90, PorMSP96) (Figure 9). Alignment of known *Larroussius* and *P. orientalis* PpSP15-like proteins revealed high degree of divergence (overall identity 24%) (Figure S3). Comparison of *P. orientalis* and other *Larroussius* species PpSP15-like proteins within each clade showed identity ranging from 61 to 96%. Our results comply well with previous reports [26], [28], [31], [56], where PpSP15-like proteins of various sand fly species were described as extremely variable proteins, likely occurring in multiple gene copies [101].

PpSP15-like proteins belong to the odorant-binding protein family but, so far, the exact function of these proteins in sand flies remains unknown. However, SP15 protein from *P. papatasi* saliva (AAL11047) was shown to elicit specific humoral and cellular immunity, which resulted in the protection of immunized mice against *Leishmania major* infection [7], [9]. Similarly, a DTH reaction was also observed in mice immunized by the inoculation of a *P. ariasi* DNA plasmid coding for SP15-like salivary protein (AAX56359) [27]. On the other hand, DNA plasmids coding for *L. longipalpis* SL1 protein (AAD32197) failed to promote the cellular immunity in experimental mice [12], hamsters [8], and dogs [68]. Glycosylation prediction servers (NetNGlyc and NetOGlyc) revealed that *P. orientalis* PpSP15-like proteins are likely not glycosylated.

### Antigen 5-related proteins

Antigen 5-related proteins (Ag5r) belong to the CAP family of proteins which is composed of **C**ysteine-rich secretory proteins, **A**ntigen 5, and **P**athogenesis-related 1 proteins. Proteins with the CAP domain are commonly present in various organisms that include prokaryotes and non-vertebrate eukaryotes [102], [103]. Ag5r proteins were described from the venom of ants, wasps and other Hymenoptera [104]–[106], but were also found in salivary glands of various bloodsucking insects, including sand flies [7], [26]–[28], [31], [50], [52]–[56]. The exact function of Ag5r in sand flies is still unknown although biological properties of other proteins from the same family may give us some clue. The X-ray structure of NA-ASP-2 protein (pathogenesis –related 1 protein) from the human hookworm, *Necator americanus*, reveals structural and charge similarities to chemokines, suggesting that these proteins could potentially modulate the host immune response [107]; more recently, a triatomine salivary member of the family was shown to have superoxide dismutase activity and to exert anti-neutrophil activity [108].

**Figure 10:**
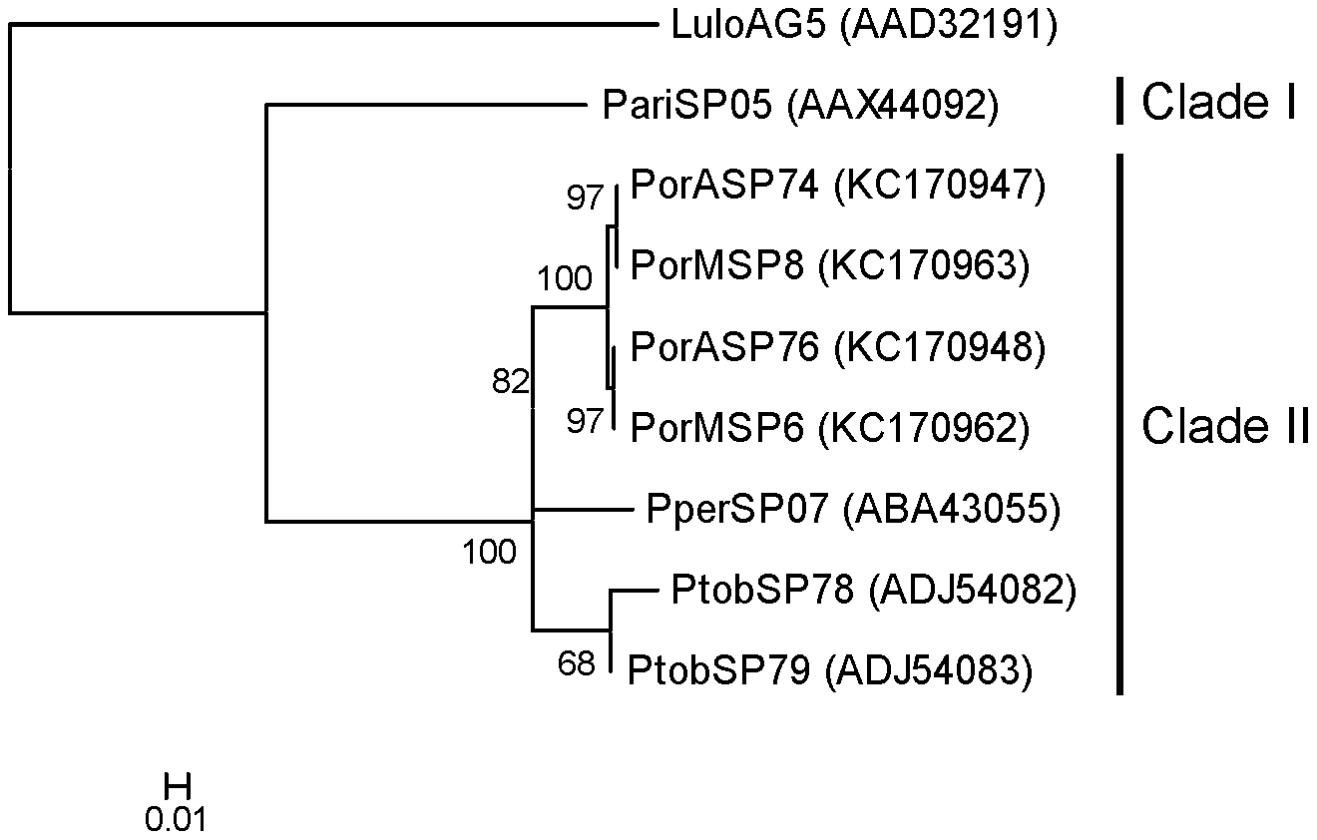
Phylogenetic analysis of the antigen 5-related family of sand fly salivary proteins. Phylogenetic analysis of antigen 5-related salivary proteins from *Phlebotomus ariasi* (Pari), *Phlebotomus perniciosus* (Pper), *Phlebotomus orientalis* Addis Zemen colony (PorA), *P. orientalis* Melka Werer colony (PorM), *Phlebotomus tobbi* (Ptob), and *Lutzomyia longipalpis* (Lulo). The Dayhoff model was used for this phylogenic analysis. Sequence names, GenBank accession numbers and branch values are indicated. Antigen 5-related proteins from *Larroussius* sand fly species are divided into two distinct clades (Clade I, II).

Sequences coding for salivary Ag5r proteins were found in cDNA library from the AZ (PorASP74/KC170947; PorASP76/KC170948) and the MW (PorMSP6/KC170962; PorMSP8/KC170963) *P. orientalis* colonies (Table 1, Table 2). The predicted molecular weight was 28.8 kDa and pI was slightly basic (8.9). Phylogenetic analysis showed that Ag5r proteins from the saliva of sand fly species from the subgenus *Larroussius* are separated into two clades (Figure 10). The first clade contains only Ag5r protein from *P. ariasi* (AAX44092), whereas the second clade includes proteins of *P. tobbi* (ADJ54082, ADJ54083), *P. perniciosus* (ABA43055), and *P. orientalis* (Figure 10). *Phlebotomus orientalis* Ag5r proteins are represented by four salivary transcripts; two from each colony (PorASP74, PorASP76, and PorMSP6, PorMSP8). Phylogenetic analysis assembled *P. orientalis* Ag5r proteins into two subclades, with an identity of 100%, the first one represented by PorASP74 and PorMSP8, the later one by PorASP76 and PorMSP6. The homology among the Ag5r proteins from different subclades reached 99% as these sequences differed in those amino acids on position 47 and 50 (Figure S4). Sequences from *P. perniciosus* (ABA43055) and *P. tobbi* (ADJ54082, ADJ54083) coding for Ag5r proteins were ascertained to be the closest relatives with the identity of 92% and 88 to 93%, respectively, while identity with *P. ariasi* protein (AAX44092) reached only 77% (Figure S4).

Antigenic properties of Ag5r proteins were demonstrated in various sand fly - host combinations. Salivary Ag5r proteins were recognized by sera of mice repeatedly bitten by *P. papatasi* [5] or *P. arabicus* [31], by sera of dogs bitten by *P. perniciosus* [63], as well as by sera of hamsters exposed to *P. tobbi* [28] or *P. argentipes* [82]. On the other hand, inoculation of DNA plasmids coding for Ag5r protein from saliva of *P. ariasi* (AAX44092) or *L. longipalpis* (AAD32191) did not elicit a specific humoral response but did induce a cell-mediated immune response [12], [27]. Glycosylation prediction servers (NetNGlyc and NetOGlyc) showed that all *P. orientalis* Ag5r proteins are N- and O-glycosylated.

### PpSP32-like proteins

The PpSP32-like protein family was described for the first time in the saliva of *P. papatasi* [7]. These proteins occur solely in sand fly saliva and their exact function is unknown. PpSP32-like proteins were found in the transcriptomes of various sand flies [7], [26]–[28], [31], [50], [54]–[56] and sequences coding for these proteins were also found in both *P. orientalis* cDNA libraries (PorASP86/KC170950; PorMSP15/KC170964) (Table 1, Table 2). The predicted molecular mass was 25 kDa and the pI was very basic (10.1–10.2). PpSP32-like proteins of AZ and MW colony revealed high degree of identity (98%); high identity was also obtained by comparing *P. orientalis* with other *Larroussius* sand fly species; 85–87% with *P. perniciosus* (ABA43053) and 81–83% with *P. tobbi* (ADJ54102). Glycosylation prediction servers (NetNGlyc and NetOGlyc) showed a high degree of glycosylation of *P.orientalis* PpSP32-like proteins, which could be potentially responsible for their immunogenicity. Sera of mice experimentally bitten by *P. papatasi* recognized *P. papatasi* SP32 protein [5] and human sera from endemic area of CL in Tunisia reacted preferentially with recombinant PpSP32 (AAL11050) prepared in mammalian expressing system [100]. On the other hand, bacterially-expressed recombinant PpSP32-like protein from *L. longipalpis* (AAS16906) did not elicit either specific humoral or cellular response [12].

### ParSP25-like proteins

Transcripts coding for ParSP25-like proteins were identified in the cDNA library from the AZ (PorASP106/KC170953) and the MW (PorMSP65/KC170972) *P. orientalis* colony (Table 1, Table 2). The predicted molecular mass was 27.6 kDa and, due to the high proportion of acidic residues present in the amino acid sequences, the pI was very acidic (4.7–4.8). ParSP25-like proteins were detected in the saliva of sand flies from the subgenus *Larroussius* (*P. ariasi*, *P. perniciosus*, *P. tobbi*), *Adlerius* (*P. arabicus*), and *Phlebotomus* (*P. papatasi*) [26]–[28], [31], [55]. ParSP25-like proteins have not yet been found in New World sand fly species [52]–[56]. The ParSP25-like proteins of AZ and MW colonies are almost identical (98%). Homology of *P. orientalis* proteins with other *Larroussius* species reached 85–86% for *P. tobbi* (ADJ54100), followed by 73–74% for *P. perniciosus* (ABA43056) and 64% for *P. ariasi* (AAX55664). Although the exact function of these proteins remains unknown, some ParSP25-like proteins were demonstrated to be immunogenic. Sera from dogs, hamsters and mice bitten by *P. perniciosus* reacted with salivary protein identified as the member of ParSP25-like family [60], [63]. Similarly to other sand fly species [28], ParSP15-like proteins of *P. orientalis* are not predicted to be glycosylated.

### Lufaxin-like proteins

A 32.4 kDa protein from *L. longipalpis* saliva belongs to a novel family of slow-tight factor Xa inhibitors, displays anti-thrombotic and anti-inflammatory activities, and is named Lufaxin (*Lutzomyia longipalpis* Factor Xa inhibitor) [109]. Members of the Lufaxin family were detected in saliva of various sand flies [26]–[28], [31], [50], [52], [54]–[56], but not in other bloodsucking insects. Sequences coding for a Lufaxin-like protein, were detected in the cDNA library of MW *P. orientalis* colony (PorMSP78/KC170976) (Table 2). Transcripts similar to Lufaxin were also found in AZ colony, but these sequences had low quality scores. The predicted molecular mass of MW Lufaxin-like protein was 18.8 kDa, the pI was 8.4. *Phlebotomus orientalis* Lufaxin-like protein was found to be highly homologous with *P. perniciosus* (ABA43054) (88% identity) and *P. tobbi* (ADJ54104) (87% identity) Lufaxin-like proteins. According to the glycosylation prediction servers (NetNGlyc and NetOGlyc) *P. orientalis* Lufaxin-like protein is N-glycosylated.

Lufaxin was previously shown to have antigenic properties. Sera of repeatedly bitten dogs recognized Lufaxin and the Lufaxin homologue from *P. perniciosus* [4], [63]. Similarly, sera of hamsters experimentally bitten by *P. argentipes* reacted with Lufaxin-like salivary protein [82]. Recombinant Lufaxin (AAS05319) was also demonstrated to promote strong cellular immunity [12], [68] and therefore was suggested as the promising candidate for vaccine against canine leishmaniasis [68].

### Other putative salivary proteins

Several other putative salivary proteins were found in both cDNA libraries from *P. orientalis* saliva. Transcripts encoding a 16 kDa salivary protein, with a pI of 5.0 and unknown function, were found in the AZ (PorASP150/KC170956) and MW (PorMSP162/KC170983) colonies (Table 1, Table 2). PorASP150 and PorMSP162 are closely related to 16 kDa salivary protein A (ACS93506) and protein B (ACS93507) from *P. arabicus* saliva. A high degree of homology was also found with salivary proteins from *P. argentipes* (ABA12153) and *P. sergenti* (ADJ54127). A related protein was recently identified in saliva of *P. papatasi* (ADJ54127). *Phlebotomus orientalis* 16 kDa proteins are likely not glycosylated.

Three clusters, encoding small salivary proteins with unknown function, were identified in each *P. orientalis* cDNA library: 3.9 kDa protein (PorASP40/KC170941; PorMSP169/KC170984), 4.9 kDa protein (PorASP68/KC170946; PorMSP196/KC170985), and 5.6 kDa protein (PorASP98/KC170952; PorMSP104/KC170980) (Table 1, Table 2). The proteins had small predicted molecular mass (3.9–5.6 kDa) and basic pI (9.2–11.0). *Phlebotomus orientalis* 3.9 kDa protein and 4.9 kDa protein were found to be closely related to the 3.7 kDa (ADJ54106) and 5 kDa (ADJ54105) *P. tobbi* proteins, respectively. Transcripts coding for a 5.6 kDa *P. orientalis* proteins share predicted sequence homology with the 4.5 kDa protein of *P. tobbi* (ADJ54097), 7 kDa protein of *P. perniciosus* (ABA43060), and the 5 kDa protein of *P. ariasi* (AAX55658). Based on the glycosylation prediction servers (NetNGlyc and NetOGlyc) we found that all *P. orientalis* small salivary proteins are likely O-glycosylated.

### Antigens and glycoproteins

**Figure 11:**
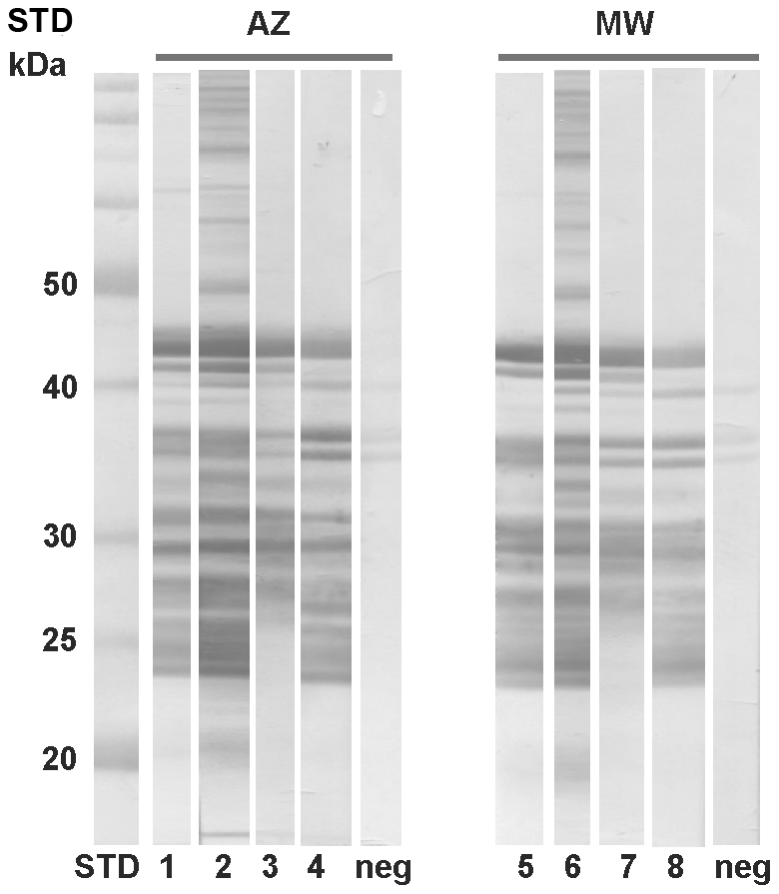
Humoral response to salivary gland antigens of Addis Zemen and Melka Werer *Phlebotomus orientalis* colony. Salivary proteins of Addis Zemen (AZ) and Melka Werer (MW) *P. orientalis* colony were separated under non-reducing conditions by SDS-PAGE electrophoresis. Western blot analysis was performed by two different sera of BALB/c mice experimentally bitten by AZ (the same sera used in the lanes 1, 5, and 2, 6) and two sera of mice bitten by MW (the same sera used in the lanes 3, 7, and 4, 8) colony. Serum from a naive mouse was used as the negative control (Neg). Molecular weight standard (STD), stained by amido black, labeled with the corresponding molecular weights (kDa).

To identify the salivary antigens in both *P. orientalis* colonies and the degree of cross-reactivity between them, electrophoretically separated salivary proteins of each colony were incubated with sera from mice experimentally bitten by either the AZ or MW colony. By comparing the western blot analysis with the *P. orientalis* proteomes (Figure 1), we predict that the most intensive reactions detected the yellow-related proteins (AZ: PorASP2, PorASP4; MW: PorMSP23, PorMSP24), apyrases (AZ: PorASP11, PorASP14, PorASP15; MW: PorMSP3, PorMSP4), and antigen 5-related proteins (AZ: PorASP74, PorASP76; MW: PorMSP6, PorMSP8). All these proteins were recognized by all AZ and MW mice sera tested, while D7-related proteins (AZ: PorASP48, PorASP122; MW: PorMSP28, PorMSP38, PorMSP67) and PpSP15-like proteins (AZ: PorASP28, PorASP37, PorASP61; MW: PorMSP12, PorMSP74, PorMSP96) were recognized only by some sera (Figure 11). Strong cross-reactivity was detected between AZ and MW *P. orientalis* colonies. The small differences in the intensity of reaction or the number of recognized protein bands were probably caused by the individual variability between mice. These data suggest that the salivary proteins in both colonies share similar antibody epitopes.

**Figure 12:**
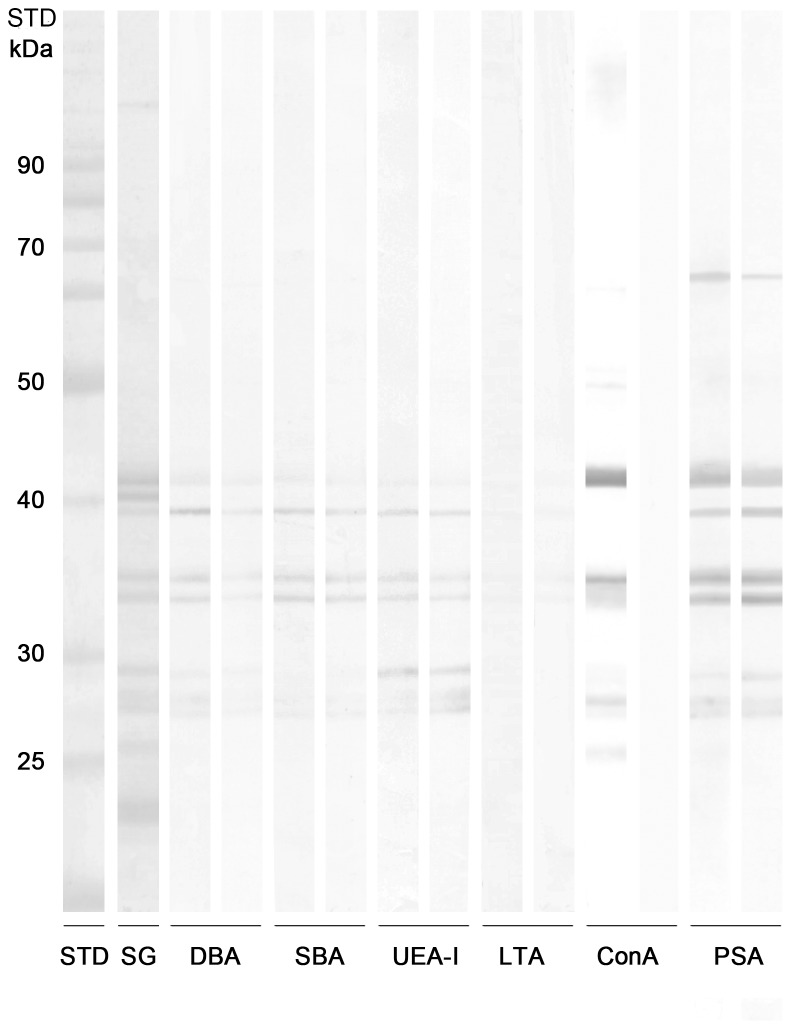
*Phlebotomus orientalis* salivary gland glycoproteins. Salivary proteins of Melka Werer *P. orientalis* colony (SG) were separated under non-reducing conditions by SDS-PAGE electrophoresis and incubated with biotinylated lectin from *Dolichos biflorus* (DBA), *Glycine max* (SBA), *Ulex europaeus* (UEA-I), *Tetragonolobus purpureas* (LTA), *Canavalia ensiformis* (ConA), and *Pisum sativum* (PSA). Doublets were used to test reactivity of each lectin; the first line represents the reaction of lectin with SG, in the later one the lectins were pre-incubated with the appropriate saccharide inhibitors to prove the specificity of reaction. Molecular weight standard (STD), stained by amido black, labeled with the corresponding molecular weights (kDa).

Due to the near predicted amino acid sequences, we chose only MW colony to study the level of glycosylation of *P. orientalis* saliva. Separated MW salivary proteins were incubated with biotinylated lectins (DBA, SBA, UEA-I, LTA, ConA, PSA) to detect mainly the N- and O-glycosylation sites. To control the specificity of the reactions each lectin was preincubated with the appropriate saccharide inhibitor. The specific reaction was observed only with ConA, the other lectins did not bind specifically or they possessed higher affinity for the glycoprotein, than for the saccharide inhibitor. We detected the specific binding of ConA to the protein bands corresponding to the 42 kDa yellow-related protein (PorMSP24), 36 kDa hyaluronidase (PorMSP108), 33 kDa salivary apyrase (PorMSP4), 29 kDa antigen 5-related salivary proteins (PorMSP6, PorMSP8), and 27 kDa D7-related salivary protein (PorMSP67), suggesting that these proteins are N-glycosylated (Figure 12). In accordance with the NetNGlyc glycosylation prediction server, the strongest reaction was detected with salivary hyaluronidase and yellow-related protein indicating that these proteins are the most glycosylated.

### Conclusions

The parasites from the *Le. donovani* complex can cause lethal VL with approximately 60 000 new cases per year [110]. Therefore, it is crucial to continue the search for the salivary proteins in relevant vector species in order to find suitable candidates of anti-*Leishmania* vaccines or markers of host exposure to sand flies. Our study provides the first detailed description of the salivary proteins of *P. orientalis*, the most important vector of VL in Northeast Africa. We made a broader comparison of the salivary gland transcriptomes, proteomes, and enzymatic activities of salivary hyaluronidase and apyrase of two laboratory reared *P. orientalis* colonies originating from an endemic focus of VL, Addis Zemen, and from a non-endemic area, Melka Werer, Ethiopia.

We revealed a high degree of homology between the AZ and MW transcripts with the overall identity of the appropriate sequences ranging from 94 to 100%. As the mitochondrial genes Cyt b and CO-I, commonly used for the molecular identification of species, reached 100% identity in these *P. orientalis* colonies [24], we assume that the slight differences in both cDNA libraries are due to the faster evolution in the genes coding for the salivary proteins. Thus, we do not consider the differences in AZ and MW colony as significant ones. Moreover, the absence of some transcripts in any of the cDNA libraries could be likely caused either by the low quality of some sequences or by the low occurrence of the transcripts in the number of randomly sequenced phages. Importantly, the equivalence of compounds and properties in AZ and MW *P. orientalis* salivary glands was also supported by the equal proteomes and enzymatic activities as well as by the powerful antigenic cross-reactivity.

Our data suggests that the composition of the salivary glands is likely not responsible for the different epidemiology of leishmaniases caused by *Le. donovani* observed in Addis Zemen and Melka Werer, although we are aware that we did not quantitatively compared the expression of various salivary proteins. Furthermore, recent study showed that also the susceptibility of both colonies to *Le. donovani* infection is identical [24]. Therefore, we can assume that there are likely other factors affecting the circulation of *Leishmania* parasites causing VL in these foci. In East Africa, the transmission and the life cycle of *Le. donovani* is not fully understood and several wild animals are suspected of being zoonotic reservoir hosts [20]. Thus, we can not exclude the possibility that the presence of putative reservoir hosts in Addis Zemen and their absence from Melka Werer may explain the different epidemiology.

Our study expanded the knowledge of the salivary proteins of sand fly species from the subgenus *Larroussius* and confirmed that *P. orientalis* is closely related to *P. tobbi* and *P. perniciosus*, two vectors causing *Le. infantum* derived CL and VL, respectively [111]. On the other hand, phylogenetic analysis determined *P. ariasi*, an important vector of visceral *Le. infantum* infection, as the evolutionarily more distinct species. Importantly, a similar relationship of *Larroussius* sand fly species was also achieved in previously published studies showing that *P. ariasi* is a more phylogenetically distinct member of the subgenus using the nuclear and mitochondrial genes ITS2, EF-α, or Cyt b [112], [113].

Overall, *P. orientalis* salivary proteins identified by transcriptome and proteome analysis can be further tested in order to explore their biological and pharmacological properties and to find out whether these salivary proteins could, in the recombinant form, be the suitable vaccine candidates. The identification of the antigenic properties of salivary proteins in several sand fly species would also indicate the feasibility of cross-protection between closely related and more distant sand fly species as promisingly demonstrated by [11], [12]. Furthermore, the humoral immune response elicited by the powerful salivary antigens would allow us to predict the intensity of exposure to sand fly bites [5], [15], [62], [63], [114] and, consequently, to estimate the risk of *Leishmania* transmission in hosts bitten by sand flies in endemic areas [15], [63], [65], [67], [115]–[117].

## Supporting Information

Figure S1 **Multiple sequence alignment of the sand fly apyrase protein family.** Multiple sequence alignment of salivary apyrases from *Phlebotomus ariasi* (Pari), *Phlebotomus perniciosus* (Pper), *Phlebotomus orientalis* Addis Zemen colony (PorA), *P. orientalis* Melka Werer colony (PorM), *Phlebotomus tobbi* (Ptob), and *Lutzomyia longipalpis* (Lulo) was performed using Clustal X 2.0. Sequence names and the number of amino acids per line are indicated. Identical amino acid residues are highlighted black and similar residues grey.
(TIF)

Figure S2 **Multiple sequence alignment of the sand fly D7-related protein family.** Multiple sequence alignment of D7-related salivary proteins from *Phlebotomus ariasi* (Pari), *Phlebotomus perniciosus* (Pper), *Phlebotomus orientalis* Addis Zemen colony (PorA), *P. orientalis* Melka Werer colony (PorM), *Phlebotomus tobbi* (Ptob), and *Lutzomyia longipalpis* (Lulo) was performed using Clustal X 2.0. Sequence names and the number of amino acids per line are indicated. Identical amino acid residues are highlighted black and similar residues grey.
(TIF)

Figure S3 **Multiple sequence alignment of the sand fly PpSP15-like protein family.** Multiple sequence alignment of PpSP15-like salivary proteins from *Phlebotomus ariasi* (Pari), *Phlebotomus perniciosus* (Pper), *Phlebotomus orientalis* Addis Zemen colony (PorA), *P. orientalis* Melka Werer colony (PorM), *Phlebotomus tobbi* (Ptob), and *Lutzomyia longipalpis* (Lulo) was performed using Clustal X 2.0. Sequence names and the number of amino acids per line are indicated. Identical amino acid residues are highlighted black and similar residues grey.
(TIF)

Figure S4 **Multiple sequence alignment of the sand fly antigen 5-related protein family.** Multiple sequence alignment of antigen 5-related salivary proteins from *Phlebotomus ariasi* (Pari), *Phlebotomus perniciosus* (Pper), *Phlebotomus orientalis* Addis Zemen colony (PorA), *P. orientalis* Melka Werer colony (PorM), *Phlebotomus tobbi* (Ptob), and *Lutzomyia longipalpis* (Lulo) was performed using Clustal X 2.0. Sequence names and the number of amino acids per line are indicated. Identical amino acid residues are highlighted black and similar residues grey.
(TIF)
