# Supplementary figures and images for: Comparative Analysis of Salivary Gland Transcriptomes of Phlebotomus orientalis Sand Flies from Endemic and Non-endemic Foci of Visceral Leishmaniasis
Source: PLoS Negl Trop Dis. 2014 Feb 27;8(2):e2709. doi: 10.1371/journal.pntd.0002709 (PMC3937273; doi:10.1371/journal.pntd.0002709)

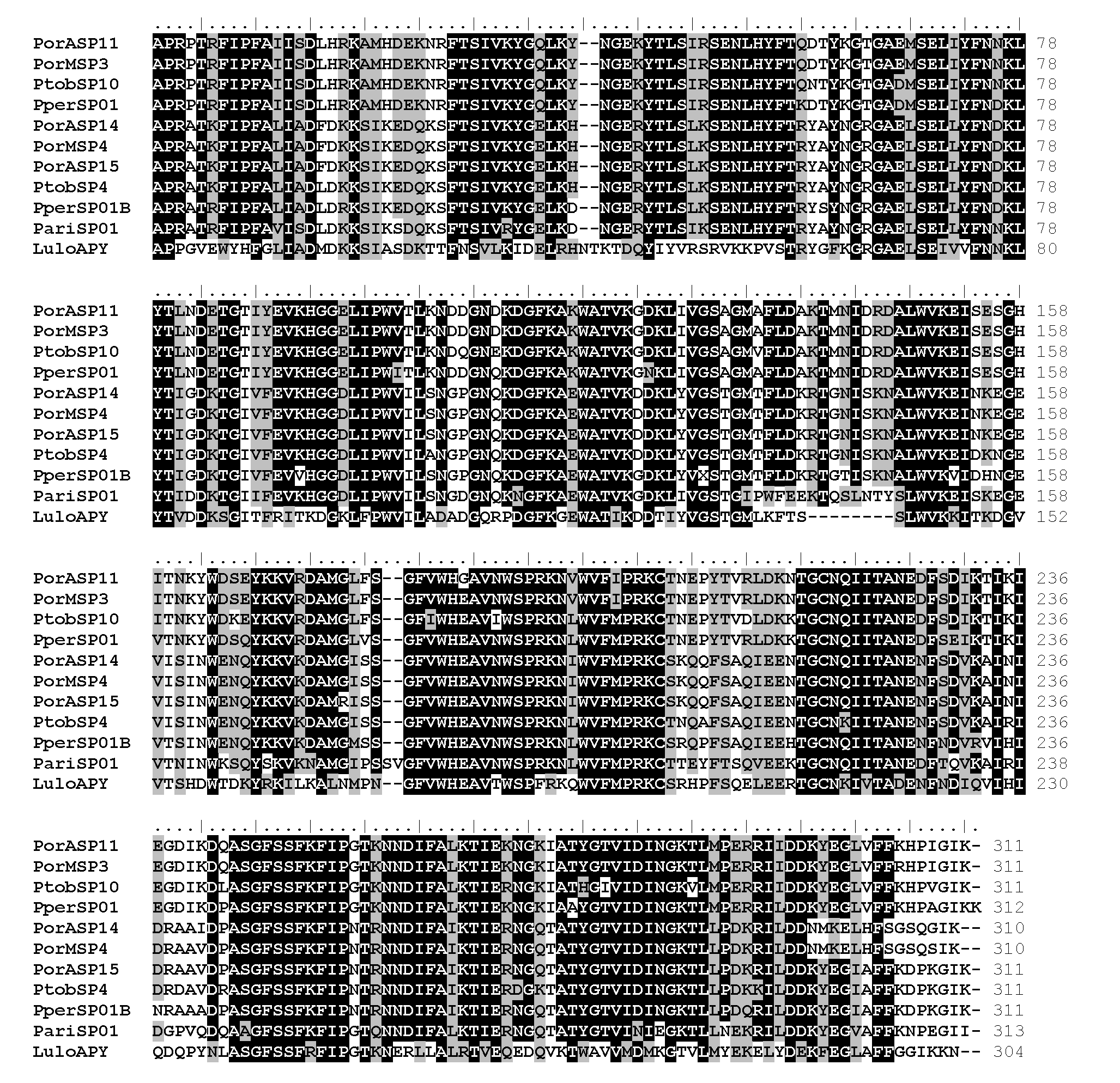

Supplement: Figure S1 — Multiple sequence alignment of the sand fly apyrase protein family. Multiple sequence alignment of salivary apyrases from Phlebotomus ariasi (Pari), Phlebotomus perniciosus (Pper), Phlebotomus orientalis Addis Zemen colony (PorA), P. orientalis Melka Werer colony (PorM), Phlebotomus tobbi (Ptob), and Lutzomyia longipalpis (Lulo) was performed using Clustal X 2.0. Sequence names and the number of amino acids per line are indicated. Identical amino acid residues are highlighted black and similar residues grey. (TIF) [file pntd.0002709.s001.tif]

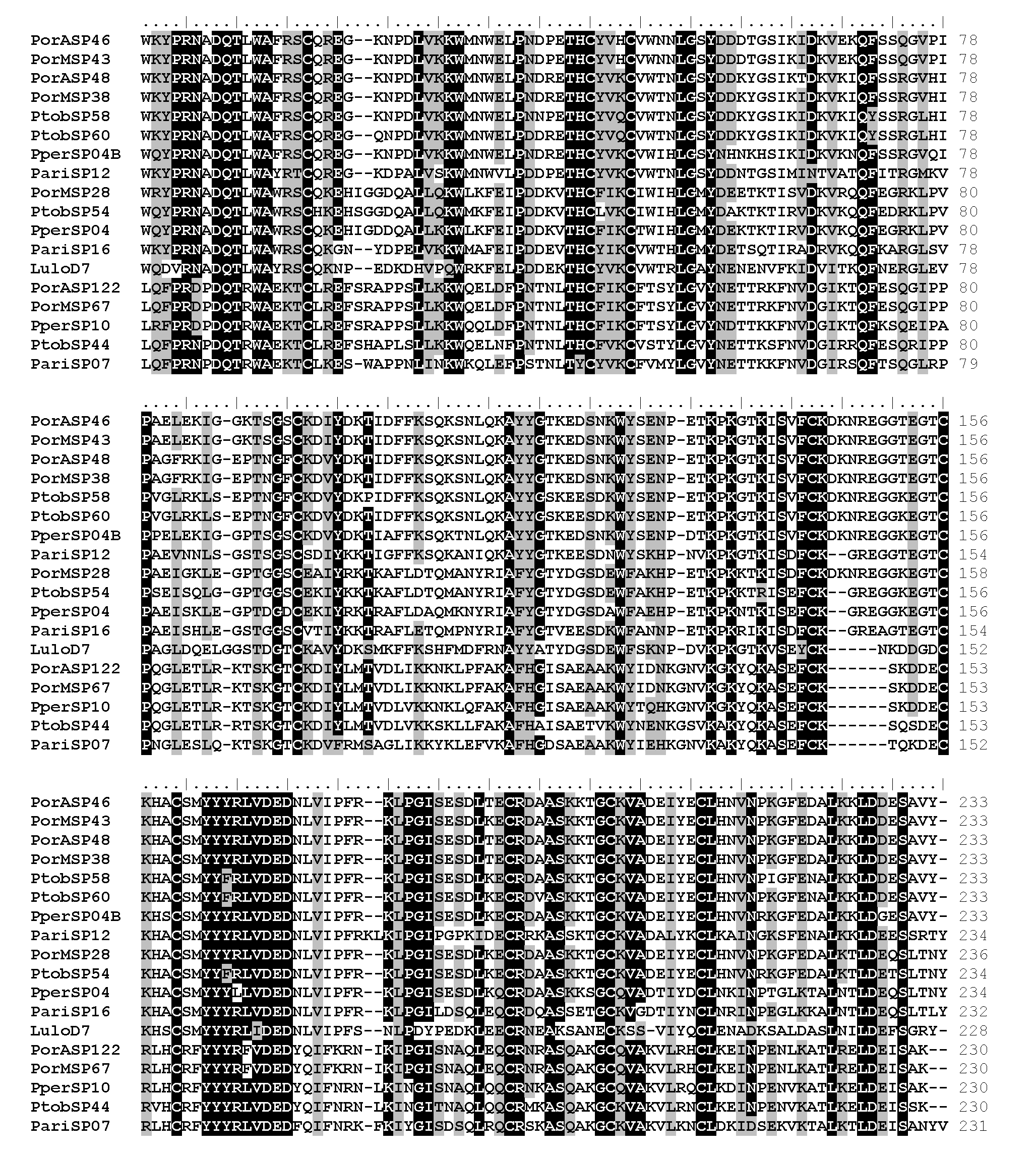

Supplement: Figure S2 — Multiple sequence alignment of the sand fly D7-related protein family. Multiple sequence alignment of D7-related salivary proteins from Phlebotomus ariasi (Pari), Phlebotomus perniciosus (Pper), Phlebotomus orientalis Addis Zemen colony (PorA), P. orientalis Melka Werer colony (PorM), Phlebotomus tobbi (Ptob), and Lutzomyia longipalpis (Lulo) was performed using Clustal X 2.0. Sequence names and the number of amino acids per line are indicated. Identical amino acid residues are highlighted black and similar residues grey. (TIF) [file pntd.0002709.s002.tif]

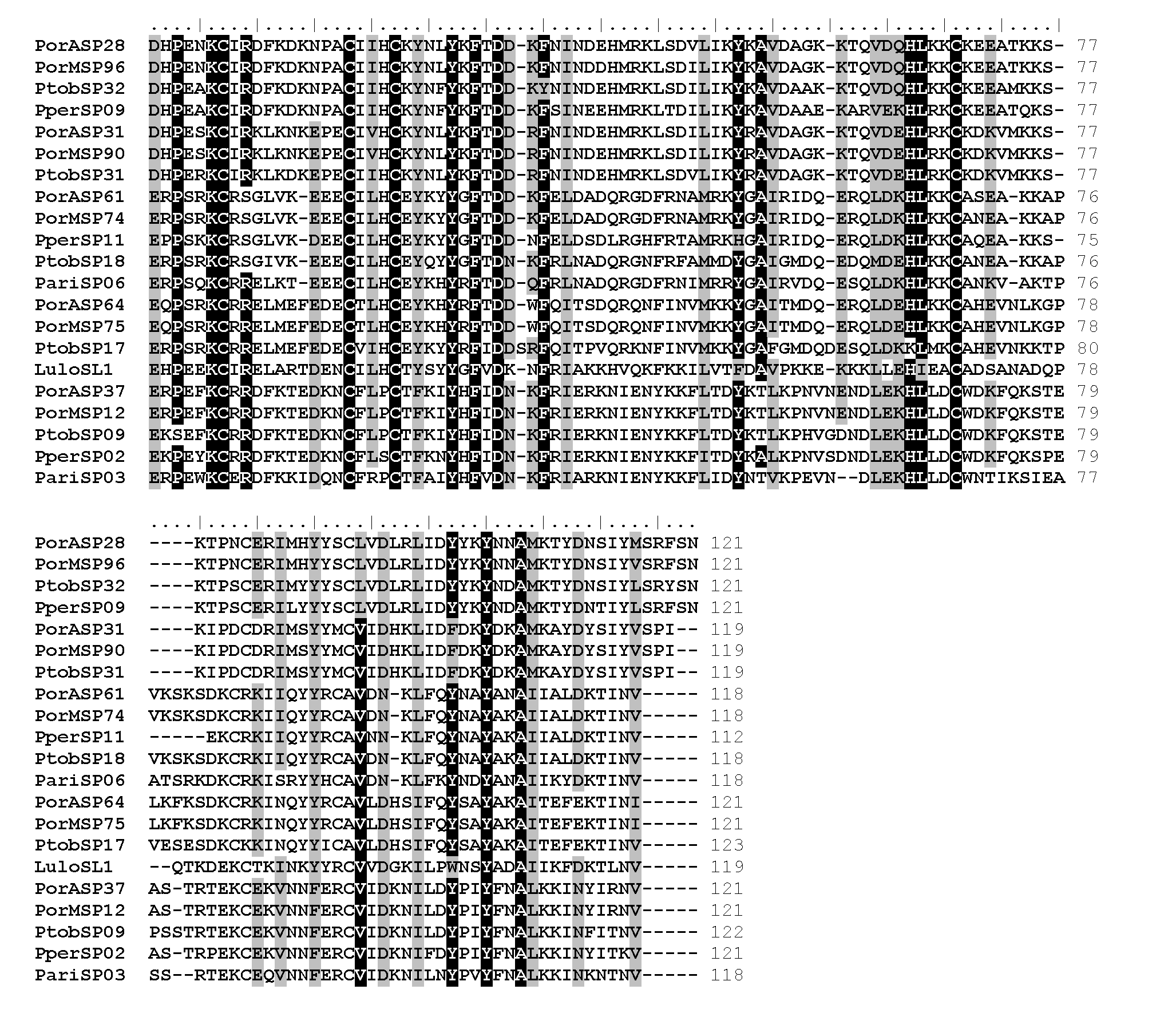

Supplement: Figure S3 — Multiple sequence alignment of the sand fly PpSP15-like protein family. Multiple sequence alignment of PpSP15-like salivary proteins from Phlebotomus ariasi (Pari), Phlebotomus perniciosus (Pper), Phlebotomus orientalis Addis Zemen colony (PorA), P. orientalis Melka Werer colony (PorM), Phlebotomus tobbi (Ptob), and Lutzomyia longipalpis (Lulo) was performed using Clustal X 2.0. Sequence names and the number of amino acids per line are indicated. Identical amino acid residues are highlighted black and similar residues grey. (TIF) [file pntd.0002709.s003.tif]

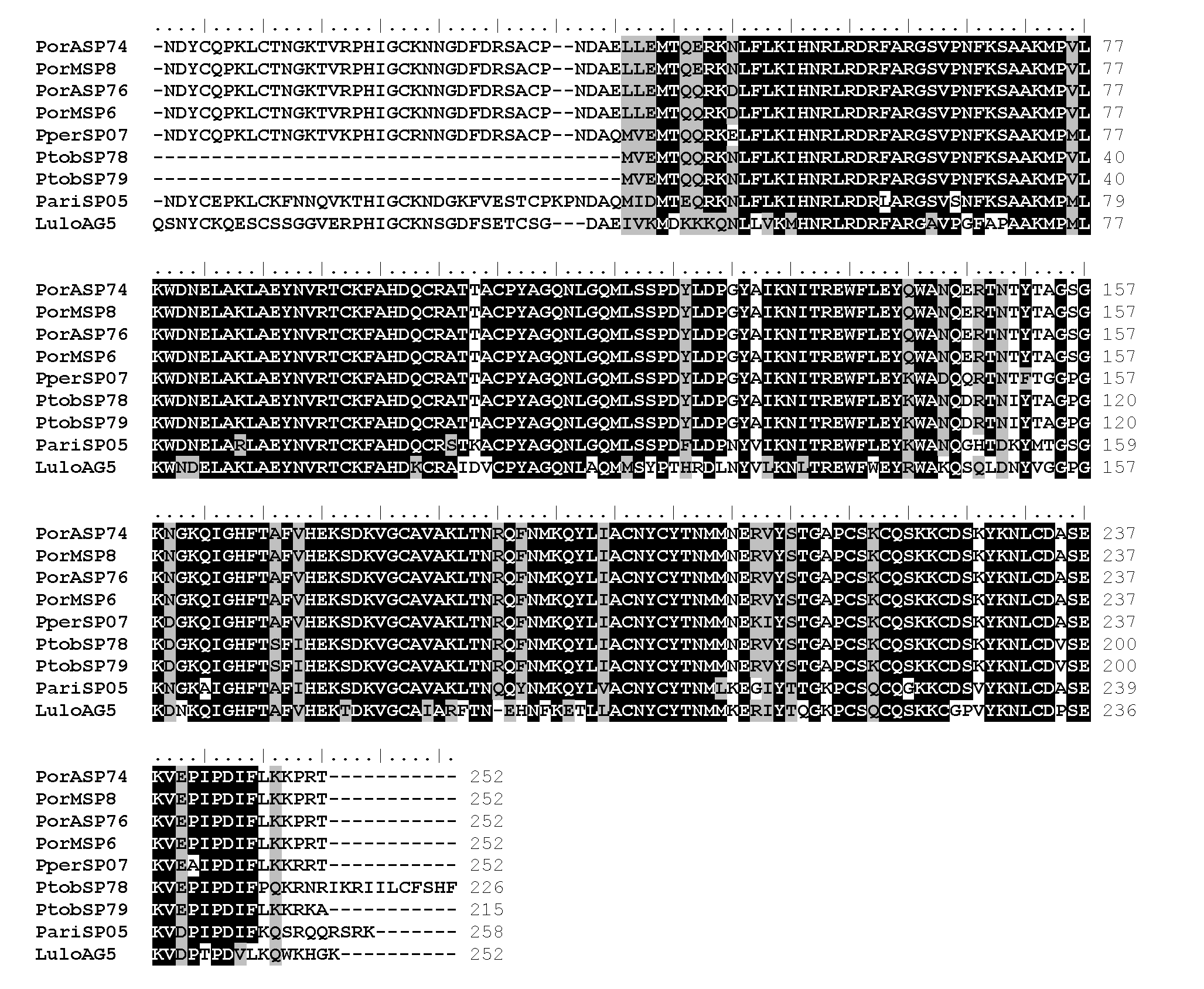

Supplement: Figure S4 — Multiple sequence alignment of the sand fly antigen 5-related protein family. Multiple sequence alignment of antigen 5-related salivary proteins from Phlebotomus ariasi (Pari), Phlebotomus perniciosus (Pper), Phlebotomus orientalis Addis Zemen colony (PorA), P. orientalis Melka Werer colony (PorM), Phlebotomus tobbi (Ptob), and Lutzomyia longipalpis (Lulo) was performed using Clustal X 2.0. Sequence names and the number of amino acids per line are indicated. Identical amino acid residues are highlighted black and similar residues grey. (TIF) [file pntd.0002709.s004.tif]
